# A Translational Approach to Increase Pulse Intake and Promote Public Health through Developing an Extension Bean Toolkit

**DOI:** 10.3390/nu15194121

**Published:** 2023-09-24

**Authors:** Chelsea Didinger, Marisa Bunning, Henry Thompson

**Affiliations:** 1 Department of Food Science and Human Nutrition, Colorado State University, Fort Collins, CO 80523, USA; chelsea.didinger@colostate.edu (C.D.); marisa.bunning@colostate.edu (M.B.); 2 Cancer Prevention Laboratory, Colorado State University, Fort Collins, CO 80523, USA

**Keywords:** beans, pulses, legumes, public health, nutrition education, Extension, information-motivation-behavioral skills model, translational research

## Abstract

Practical, affordable solutions need to be implemented to address global challenges confronting human and environmental health. Despite a myriad of benefits for people and the planet, beans and other pulses (e.g., chickpeas, cowpeas, dry peas, lentils) are under-consumed. To better understand consumer concerns and interests, a Food Habits Survey was conducted and the findings were incorporated into the Colorado State University Extension Bean Toolkit. Guided by the Information-Motivation-Behavioral Skills model, the toolkit included informational social media posts, cooking guidance, and an online class. A convenience sample of participants was recruited through Extension and university networks. After class participation, significant gains in knowledge of pulse nutrition, versatility, and cooking were observed, with an average increase of 1.5 points on a 5-point Likert scale (*p* < 0.001). Moreover, participants (*n* = 86) perceived a greater importance of motivators (e.g., nutrition, versatility, environmental benefits) and found barriers (e.g., flatulence, long cooking times, unfamiliarity) to be less discouraging. Most participants reported an intention to eat more pulses, and among those who completed the 1-month follow-up survey, pulse intake frequency increased (*p* = 0.004). Emphasizing motivating factors while simultaneously mitigating barriers to consumption can help reverse insufficient intake and promote healthy behavior change. Leveraging Extension or similar networks is one way to adopt a translational approach to better reach the public with this information.

## 1. Introduction

The world is facing severe challenges for human and environmental well-being, including a cost-of-living crisis, rising rates of obesity and chronic disease, and climate change [1,2,3]. Accordingly, there is dire need for affordable, accessible solutions. Beans and other pulses (i.e., the edible, dry seeds of non-oilseed legumes like chickpeas, cowpeas, dry peas, and lentils) have a relatively economical price and the ability to simultaneously promote public and planetary health [2,4,5,6]. Thus, pulses can play a key role in addressing many global challenges.

Despite the myriad of benefits they offer, consumption of pulses is low in many countries around the world. Average global consumption appears to be stagnating around only 21 g per day per capita [7]. Yet, there is evidence that consumption can be higher. For example, several countries in Eastern Africa eat around 150 g per day [8]. Indeed, an improved nutritional status appears to go hand-in-hand with higher consumption of pulses. Individuals who regularly eat more beans and other pulses have higher intake of several key dietary components (e.g., dietary fiber, folate, potassium) and also consume lower levels of fat [9,10].

The adoption of more pulse-centric eating habits can allow people to capitalize on the benefits of beans and other pulses for human and planetary health. A key approach to encourage higher pulse intake is to emphasize the motivating factors of pulses while simultaneously addressing any barriers, i.e., consumer concerns that may prevent higher pulse intake [11]. Motivators include their versatility, affordable price point, nutrient-dense profile, and contributions to healthy and sustainable eating patterns [12,13]. Detailed information about the benefits of pulses for gut health, healthy weight maintenance, and chronic disease (e.g., diabetes, cardiovascular disease, certain types of cancer) prevention is beyond the scope of this paper but can be found in the recent literature [4,14,15,16,17].

Despite numerous benefits, there are several primary barriers to higher pulse intake that are well-established in the literature, including long cooking times and concerns over flatulence [11,13,18]. Another key barrier is unfamiliarity, or a lack of understanding of how to prepare pulses and regularly include them in a variety of meals [11,19]. For example, Doma and colleagues found that consumers tend to prefer to eat beans at certain times and in particular dishes [13]. Most consumers they surveyed ate beans in the winter, for dinner, and mainly in dishes like chili and soup [13]. This presents a challenge because lack of awareness of the versatile ways to eat pulses for different meals of the day and in a wide variety of dishes can reduce the likelihood that consumers will regularly choose to include them. Thinking beyond more traditional meals like chilis and soups to varied, diverse options can provide a wider array of choices to incorporate pulses into daily diets. This could include dishes like smoothies, salads, sheet-pan bakes, and other ways to include pulses in breakfasts, lunches, snacks, dinners, and desserts.

Our research team recently conducted a citizen science project to inspire people with the culinary versatility of pulses and received participant feedback on the Bean Cuisine (i.e., a 2-week cuisine that incorporated beans and other pulses into 14 unique breakfasts, lunches, snacks, and dinners for a total of 56 recipes) [19]. Participants significantly increased their knowledge about the health benefits of pulses, pulse versatility, and how to prepare dry pulses. They also reported feedback such as, “I’m more aware of ways to incorporate beans into every meal, and I’m paying more attention and trying to eat them more” [19] (p. 12). Citizen science can be a powerful approach to engage the public [20], but it is also important to reach a broad audience that may not have the ability to participate in a citizen science project. One way to accomplish this is to leverage Extension to quickly disseminate information throughout its network. Extension, which is part of the land-grant university system, is well-connected in rural and urban areas throughout the United States, and staff lead programs and initiatives within their respective counties to improve the well-being of communities [21].

The mission of Extension is to translate research into action by bringing evidence-based discoveries and information to local communities to support public health [22]. Thus, Extension is perfectly suited to accomplish translational research. Traditional science often considered translational research as “bench-to-bedside” work that served as an interface between basic science and the clinic [23]. Yet, translational science also encompasses ensuring that research knowledge reaches the public such that they can reap the benefits of scientific findings [23]. For this type of translational science, helping individuals change behaviors and make more informed choices that promote public health is the goal. Moreover, it is not the clinic that is the research setting, but rather the community [23,24].

To conduct translational research through Extension, we developed a toolkit, or a collection of educational program resources targeting an issue [25]. This paper addresses increasing consumption of a variety of pulses—beans as well as chickpeas, cowpeas, dry peas, and lentils. However, the toolkit was called a “Bean Toolkit” instead of a “Pulse Toolkit” due to higher familiarity with the word “bean.” Nonetheless, due to including several pulses in addition to beans, the toolkit is attractive and relevant to a broad population. As it is called the Bean Toolkit, the words “pulse” and “bean” may be used interchangeably in this paper.

The Colorado State University (CSU) Extension Bean Toolkit is intended to reach a wider audience via multiple outlets of the Extension network, such as a 1-h online class, handouts, social media posts, and other creative outreach tools with information about beans. Before creating the toolkit, a Food Habits Survey was designed and conducted. Survey findings were combined with results from the scientific literature to ensure consumers’ interests and concerns would be addressed. The CSU Extension Bean Toolkit was designed to mitigate established consumer barriers and emphasize motivators. Doing so promotes increased intake of beans and other pulses, which has the potential to advance the health of both people and the planet.

## 2. Materials and Methods

### 2.1. Food Habits Survey Development

To inform the development of the Extension toolkit and class, an online survey was developed in Qualtrics. The survey was conducted to better understand consumer viewpoints, behavior, and preferences. The lead author developed the survey questions based on consultation with experts and a literature search of barriers and motivators for pulse consumption. Similar question topics and format to other surveys assessing pulse intake-related information amongst consumers were adopted, such as those by Doma [13], Heer [26], Palmer [27], and Winham [28]. Questions were refined with the other authors, who are experts in pulses and/or Extension program development and evaluation. To establish content validity, experts were asked to provide feedback on question and response option wording, content, and clarity [29,30,31]. Their areas of expertise included pulse nutrition and health benefits, consumer behavior as it relates to pulses, outreach work, survey design, and program evaluation.

After modifying the survey per expert feedback, a pilot was conducted to measure reliability, or the ability of the survey to produce consistent results [29]. To assess test-retest reliability, the survey was administered at two time points approximately 2 weeks apart (average time between responses = 14.2 days), with no intervention in between. A convenience sample of the following two groups completed the survey pilot: university students and Extension volunteers. Overall, 33 individuals completed the survey at two separate time points (*n* = 27 indicated female, *n* = 27 White, *n* = 19 were between 18–39 years of age, and *n* = 12 were between 50 and 79 years of age). Although 33 is not a large number for a test-retest approach, questions were designed to be like other Extension surveys and previous research on motivators and barriers to bean consumption. Thus, the main purpose was to ensure participant understanding of question wording and determine via free response feedback if appropriate response options had been provided. The Food Habits Survey was designed to inform toolkit and class development and was not intended to be a standalone study on consumer behavior and preferences.

For non-free response questions, Spearman correlations measured correlations between the test and retest scores for ordinal and/or Likert-type data [29]. Generally, survey items are considered reliable when they have a correlation coefficient of at least 0.7 and *p*-value of less than 0.05 [29,30]. Percent agreement was also calculated because, for small sample sizes, correlations can be misleading and agreement values are another way to assess consistency, especially for categorical variables [29,32]. Moreover, agreement is one way to examine Likert scales and determine if people are choosing similar responses (e.g., “strongly agree” at the first time point and “somewhat agree” at the second time point). Percent agreement values greater than 66% are considered fair [32].

Based on Spearman and agreement values, as well as any feedback received, questions were either removed or modified as needed, with decisions reached via researcher corroboration. The wording of all questions and response options was reviewed. Modifications or removal occurred for questions with a *p*-value greater than 0.05, for which Spearman correlation scores were below 0.7, and/or with low agreement scores. Examples of modifications include:

- **Question removal**. For example, one of the original questions asked about the elevation at which participants live, which can influence cooking time of dry pulses [33,34,35]. Participants were directed to a site to tell them their elevation. This question was removed because participants said that being redirected to another site sometimes exited them from the survey. Another question that was removed was one that asked about the importance of the barrier “concern about antinutrients like lectins,” due to low Spearman correlation and agreement scores (ρ = 0.47, *p* = 0.007, agreement = 0.56).
- **Response option removal**. For several questions, the option of “other” was removed because no one filled in this response, indicating the provided response options were sufficient. The consulted experts also did not suggest other response options.
- **Question and/or response option wording clarification**. For several questions, statements that clarified that more than one option could be selected were added.

This survey was an integral part of the formative evaluation and development of the Extension Bean Toolkit and class. The final Food Habits Survey can be found in Supplementary Materials File S1. A link that provided access to the survey was distributed via multiple platforms, including emails, newsletters, and social media posts. It was advertised as a Food Habits Survey to reduce bias with regards to bean preferences and consumption. This was similar to the approach taken by Doma and colleagues, wherein the survey was called the Food Survey Study [13]. Those who completed the Food Habits Survey could choose to receive materials developed as a result of the survey, a summary of survey findings, and to be entered into a drawing to win one of multiple $20 Amazon gift cards. This survey work was approved by the Colorado State University Institutional Review Board, protocol #2016. Survey findings informed development of the Extension Bean Toolkit components and online class.

### 2.2. Development of Toolkit Components

The components of the CSU Extension Bean Toolkit were developed based on the findings from the Food Habits Survey, a review of the literature, and input from experts and Extension colleagues. For instance, our recent study on how elevation and cooking conditions impact cooking times provides a summary of tips to shorten cooking times [35]. The tips and handout that were developed as part of that research were provided to consumers via the Extension Bean Toolkit. Ways to reduce cooking time include soaking, adding salts to the soaking water, and utilizing fast-cooking pulses (e.g., lentils) [35,36,37,38]. Regarding flatulence, the literature shows that this concern is overexaggerated [39], a point that is important to relay to consumers. Practical suggestions to reduce flatulence include slowly increasing consumption of pulses [39] and discarding the soaking water if preparing dry pulses in the home [40]. Unfamiliarity is another key barrier to address. The literature suggests that recipes are one way to accomplish this [41]. Therefore, unfamiliarity was mitigated through providing a range of simple, creative, and tasty recipes. Information to equip consumers to overcome these barriers was provided in the Extension class and toolkit.

The purpose of the toolkit was to educate consumers on the benefits and versatile uses of pulses and provide accessible cooking tips, thereby helping to increase knowledge and awareness of pulses. The Information-Motivation-Behavioral Skills (IMB) model guided toolkit development. The IMB model states that there are three core determinants of the performance of health behaviors: (1.) information that can be translated into behavior; (2.) social and personal motivation; and (3.) behavioral skills that facilitate the behavior [11,42,43]. The Extension Bean Toolkit appealed to all three constructs of the IMB model. Information was provided about the many benefits of pulses and how to prepare them. Personal motivation was appealed to through explaining the desired health benefits that can be obtained through regular pulse consumption. Social motivation occurred through creating a community of learners in the online class and encouraging them to engage with one another via the chat feature. Improved behavioral skills were promoted via in-depth explanations of how to prepare pulses and the various ways of using them, and through providing handouts and recipes. The main toolkit components included:

- **A bean calendar**. The bean calendar, Bean Appetit, had an introduction with background about pulses and practical cooking information. Each month contained a photo, a short caption, and a QR code that linked to a recipe and/or other related page with helpful pulse information. The web pages are available on Food Smart Colorado, an affiliate website of CSU Extension that provides nutrition, health, and food safety information. As part of the calendar, 13 pulse-centric recipes were developed, and they can be found on the website: https://foodsmartcolorado.colostate.edu/food/2022-bean-calendar-recipes/.
- **Social media**. Monthly social media posts were designed and posted from January 2022 to October 2023. The first year of posts were associated with the months of the bean calendar. The second year of posts addressed other topics of consumer interest, such as human and environmental health benefits, information about how to cook beans, and creative recipe ideas. Regular posting allowed followers of Food Smart Colorado to be exposed to various types of information that addressed potential barriers to pulse intake and emphasized motivators. Analytics such as viewer reach were assessed through Food Smart Colorado’s Facebook Professional Dashboard.
- **Handouts**. Two main CSU Extension handouts were developed, one titled “Cooking Dry Beans” and the other “Tips for Cooking with Dry Beans and Other Pulses.” The handouts were distributed as part of the Extension class, as well as being made available online at Food Smart Colorado and linked to in social media posts.
- **Blog posts**. The lead author wrote several pulse-related blogs on Live Smart Colorado, a CSU Extension blog run by Family & Consumer Science Extension Agents and Specialists at CSU.

### 2.3. Designing and Conducting the Extension Class

Class topics were informed by areas of consumer interest and motivators and barriers for pulse consumption, as determined by the Food Habits Survey and a review of the literature [11,12,13]. To structure the class, a farm-to-table approach was decided upon. The class began with discussions of bean crops in the field and their sustainability benefits, followed by nutrition and health information, practical cooking tips, and creative and delicious ways to regularly incorporate beans into the diet. For all topics, motivating factors were emphasized, and potential areas of misinformation and barriers were addressed.

The 1-h online class was offered through Zoom and was promoted through the Extension network, colleagues, CSU emails and newsletters, and social media. All surveys were designed in Qualtrics. Questions came from previous Extension class surveys and the questions that had been piloted and assessed for validity and reliability during development of the Food Habits Survey. This Extension work was approved by the Colorado State University Institutional Review Board, protocol #3589.

#### 2.3.1. Validation Class

After the design of the class, an initial testing of impact was conducted, herein called the validation class. The lead author worked with CSU Extension agents throughout the state to promote the class, holding six different class sessions in partnership with six different counties in Colorado. For the validation class, survey data was collected at three time points. This provided the chance to: (1.) gather more complete feedback from participants about the class to see if any changes should be made before the final class implementation; and (2.) better understand the potential impact on short-term behavior. Before the class, participants completed the pre-survey (Supplementary Materials File S2), followed by the post-survey immediately after the class (Supplementary Materials File S3). After the class, participants were emailed the CSU Extension bean handouts developed as part of the toolkit, as well as links to recipe resources on Food Smart Colorado and Colorado Dry Beans. This was part of a Colorado Department of Agriculture funded project, so this helped direct people to an organization that can provide local bean information. To assess impacts on short-term behavior, participants were also contacted to complete a 1-month follow-up survey (Supplementary Materials File S4), with the chance to receive a $10 Amazon gift card as an incentive upon completion of all three surveys.

Participant feedback was positive, and no modifications to the slides were necessary. However, a couple of points were incorporated into the final class. One, people requested more recipe information. However, there was not adequate time in the class to discuss recipes in-depth and still address other topics. Thus, it was emphasized that recipes would be shared in the follow-up email, and links were immediately sent to participants after completion of the class. Also, there appeared to be confusion about the wording of the question asking about the importance of barriers, as is discussed in the results. Therefore, the wording and response options on the final class survey were adjusted as follows:

- Original wording: “How important are the following in discouraging you from eating pulses? ‘Important’ reflects a factor that discourages you. ‘Unimportant’ represents a factor that does not discourage you.” Response options ranged from “very important” to “very unimportant.”
- Modified wording (asked twice, to assess importance before and after): “BEFORE/AFTER the class, how important were the following in discouraging you from eating pulses?” Response options ranged from “highly discourages” to “does not discourage.”

#### 2.3.2. Final Extension Class

For the final class, only one survey was administered immediately following the class. This reduced respondent burden and increased the likelihood of gathering complete data from participants, as opposed to an incomplete set of responses to a multi-survey set like in the validation class. Retrospective pre-post design has been found to afford many benefits, including requiring less time to administer, establishing a common metric, and reducing the response-shift bias that can occur with program participation [44,45]. See Supplementary Materials File S5 for the final class survey. Questions were a compilation of those on the validation class surveys. Instead of asking about knowledge level and the importance of barriers and motivators at multiple time points, participants were provided two matrices. One asked about knowledge, motivators, and barriers before the class and the second about after completion of the class. For example, participants rated their knowledge of three different topic areas (nutrition and health benefits, versatility, and how to cook dry pulses) by responding to the following two questions:

1. On a scale of 1 (low) to 5 (high), how would you rate your knowledge of the following BEFORE the class?
2. On a scale of 1 (low) to 5 (high), how would you rate your knowledge of the following AFTER the class?

In addition to CSU Extension, outside venues also hosted the class, such as the Denver Botanic Gardens. As with the validation class, participants were sent a follow-up email with the CSU Extension bean handouts and links to recipe resources on Food Smart Colorado.

### 2.4. Statistical Analyses

All statistical analyses were conducted in IBM SPSS Statistics version 28 (IBM Corp. Released 2021. IBM SPSS Statistics for Windows, Version 28.0. Armonk, NY, USA: IBM Corp.). Statistical analysis to assess test-retest reliability of the Food Habits Survey questions is detailed in the above Food Habits Survey section. When analyzing the final data set for the Food Habits Survey and the Extension class, the statistical analysis was conducted similarly to that of our recent Bean Cuisine citizen science research [19]. Briefly, descriptive statistics were used for categorical variables like demographics. For Likert scale questions about knowledge and the importance of barriers and motivators, Wilcoxon signed rank test was used to assess if significant changes occurred.

For free response data, the lead author utilized an inductive approach, beginning with opening coding [46,47]. All free response data was reviewed to create categories and subcategories, which were updated as the analysis progressed [13,48,49]. Via comparative analysis, related codes were categorized and themes were extracted. The constructs of the IMB model [42] informed theme extraction, with focus on key information, motivational aspects, and behavior changes that could influence pulse intake [11]. These codes were grouped into themes that were fully supported by the data and corroborated with the other researchers on the project. Quotes were then selected to illustrate the themes that emerged and arranged into tables.

## 3. Results

### 3.1. Food Habits Survey

A total of 940 individuals completed the Food Habits Survey (see Supplementary Materials File S1 for the survey questions). There was higher participation by White, educated females: 82.8% of participates selected White as their ethnicity, nearly 33% had a master’s degree, and about 80% were female. A relative lack of socioeconomic diversity is a challenge faced in other outreach work [19]. Although this could limit generalizability to a larger audience [20,50], it may be representative of the population likely to utilize the resulting Extension Bean Toolkit resources and take the Extension class. This is because the recruitment methods were similar for both, thus a comparable group is likely to be reached when these resources are promoted. For more details about respondent demographics, see Supplementary Materials File S6.

#### 3.1.1. Pulse Intake Frequency and Preparation Habits

When asked about frequency of pulse intake, the most frequent response was “1–3 days per week” (*n* = 438, or 46.6%), as shown in Table 1. When “never” was coded as 1 and “every day” as 6 (i.e., higher numbers corresponded to more frequent intake), average consumption was 4.1, with a standard deviation of 0.94. Notably, this survey question only provided data on a range of frequency of consumption, not the exact number of days on which pulses were consumed, nor the intake volume. Thus, the average consumption score of 4.1 represents a range of days per week that the average survey respondent eats pulses, but it does provide more detailed information on average intake. This is a limitation of the survey tool, as will be discussed later in more detail.

Most participants reported that they were omnivores (*n* = 680, 72.3%), followed by pescatarians (*n* = 106, 11.3%), vegetarians (*n* = 84, 8.9%), and vegans (*n* = 69, 7.3%). When examining frequency of pulse intake by dietary pattern, it is evident that those respondents who follow pescatarian, vegetarian, and vegan dietary patterns eat pulses more often than the omnivore group. As shown in Table 2, vegans were the group with the highest frequency of pulse intake. Whereas some omnivores (*n* = 4) selected never for pulse intake frequency, the least frequent option selected by pescatarians was “several days per year, but less than 1 day per month.” For both the vegetarian and vegans surveyed, the least frequent option selected was “1–3 days per month.” This suggests that these groups demonstrate more frequent pulse consumption than omnivores.

Participants provided information about their pulse cooking and consumption habits. The majority of individuals (*n* = 882, ~94%) had cooked with dry pulses at least once before. This is a high percentage and may represent a bias or limitation. However, the question only asked whether participants had cooked dry pulses at least once. Thus, it is not clear what percentage of these 882 respondents regularly cook dry pulses versus how many have only cooked dry pulses once or a few times. When asked “Which cooking method(s) do you regularly use when preparing beans? You can select more than one,” the highest number of respondents selected stovetop (*n* = 594 of the 882 that have cooked dry pulses before), followed by slow cooker (*n* = 350) and electric cooker (*n* = 330). Traditional pressure was the least common response (*n* = 93), with more people regularly using their ovens (*n* = 155) than a traditional pressure cooker.

Soaking dry pulses was a common practice among those who cook dry pulses. Only 63 of the 882 people who cook dry pulses indicated that they never soak before cooking. The most common response option when asked “How likely are you to soak dry beans and other pulses before cooking?” was “Very often (81–100% of the time)” (*n* = 230). Of the respondents who soak dry pulses at least some of the time before cooking, most people discard the soaking water (*n* = 625), with a much smaller number that chooses to cook in the soaking water (*n* = 188). Additionally, *n* = 374 individuals (about 46% of those who indicate they soak dry pulses) have tried the quick soak method. This method entails boiling for about 3 min and then letting stand for 1 h before cooking, instead of a longer overnight soak [35,51].

Respondents were also asked about what influenced their decision to use salt when cooking with dry pulses. This question was asked to the 882 individuals who indicated they have cooked dry pulses before. It was evident that there is confusion about this topic, because almost the same number of people indicated that salt can help pulses soften (*n* = 139) as did the number that thought salt prevents pulses from softening (*n* = 137). Therefore, addressing this potential area of confusion could be a helpful topic to cover in consumer resources and class. Overall, the most common factor that influenced the decision to use salt or not was recipe instructions (*n* = 237), indicating the importance of clear, well-written recipes.

#### 3.1.2. Pulse Preference and Form in Which Pulses Are Eaten

Respondents were asked about how much they liked five main types of pulses, with results shown in Table 3. Dry beans were the favorite type of pulse (*n* = 803 like dry beans), followed by chickpeas (*n* = 766). Cowpeas (also called black-eyed peas) and dry peas were the least favorite type of pulses, with *n* = 139 and *n* = 132, respectively, indicating they do not like these pulses. Cowpeas also appeared to be the least familiar pulse to respondents, with the highest number of individuals (*n* = 89) indicating they have not tried them before.

Participants also indicated how they have eaten pulses in the last year. They were provided with a list of types of pulse dishes and could select multiple options. Table 4 indicates the breakdown of how respondents reported eating pulses. Pulses mixed with grains, dips, and soups all had over 700 respondents indicate that they have eaten pulses this way within the last year, with nearly 700 selecting chili. Dishes made using pulse flour were less common ways to consume pulses. Dessert was by far the least common way people ate pulses, with only 192 individuals indicating they have eaten desserts with pulses within the last year.

#### 3.1.3. Motivators and Barriers to Pulse Intake

Respondents also indicated the relative importance of various motivating factors to eat beans. Table 5 shows how respondents rated the importance of nutritional factors of beans. As shown in the table, protein and fiber were deemed the most important nutritional factors by respondents. The total number of important responses (i.e., the sum of “very important” and “somewhat important”) was similar for fiber and protein, *n* = 809 and *n* = 812, respectively. The average importance score of protein was slightly higher than that of fiber though, also with a smaller standard deviation. Although the low-calorie nature of beans and other pulses was still important to respondents, it ranked as the least important of the nutritional motivators.

Table 6 lists other motivational reasons for eating pulses, beyond the nutritional factors shown in Table 5. Based on the results shown in Table 6, taste was the most important factor for survey participants, with nearly 91% indicating this was important. Taste was followed by health benefits, with almost 85% indicating this was important to them. The affordable price point of pulses and their environmental and sustainability benefits were rated as similarly important, with average ratings of 3.99 and 3.98, respectively. Gluten-free ranked dramatically less important compared to other factors, with the most common response option selected being “very unimportant.”

When asked about potential barriers to pulse intake, the most important barriers were long cooking times (with *n* = 472, 50.5% ranking this as important), concerns about flatulence (*n* = 366, 39.0%), and being unsure how to prepare pulses (*n* = 287, 30.8%). However, after conducting the Extension validation class, it was determined that the wording of this question and response options were confusing to participants. The question asked: “How important are the following in discouraging you from eating pulses? ‘Important’ reflects a factor that discourages you. ‘Unimportant’ represents a factor that does not discourage you.” Needing to rank the importance of barriers as important is similar to a double negative structure, and this appeared to confuse some individuals. Therefore, results of the ranking of importance of barriers are not shown in detail here. More detailed responses about the importance of certain barriers can be found in the results of the final Extension class, after question wording was modified for improved clarity.

A question about barriers to cooking with dry pulses was also asked, but in a different format. Respondents were asked whether “any of the following ever prevented you from cooking with dried pulses? Please select all that apply.” Response totals are shown in Table 7. The numbers align with the literature, indicating that long cooking times can pose a major barrier to pulse consumption.

#### 3.1.4. Topics of Interest and Preferred Resource Format

The main purpose of the survey was to use findings—in conjunction with expert input and the literature review—to inform the development of the Extension class and toolkit resources. Thus, respondents were asked about topics of interest and preferred formats. Nearly 68% of respondents indicated interest in electronic handouts and 41% in printed resources. Accordingly, handouts were developed to be posted and distributed in an online format, but formatted as a PDF such that they could easily be printed. The percentage of people who indicated interest (i.e., the sum of those who indicated very or somewhat interested) in the following topics was as follows: (1.) 74.3% indicated interest in tips to include more pulses in their diets; (2.) 62.5% in myth busting (i.e., addressing myths and misinformation related to pulses); (3.) 61.0% in nutritional information; (4.) 54.5% in flatulence and ways to address this concern; and (5.) 48.9% in the effects of elevation on the cooking of dry pulses. As many participants either did not regularly cook dry pulses and/or did not live at high elevation, it is reasonable that this topic would be of the least interest.

Most individuals indicated that they would prefer an online class (*n* = 555, 59.0%) to an in-person class (*n* = 136, 14.5%), and *n* = 231 (24.6%) selected no preference. Due to this response and the ability to reach audiences with a wider geographical spread, it was decided to conduct the class online.

### 3.2. CSU Extension Bean Toolkit

#### 3.2.1. Bean Calendar

**Figure 1:**
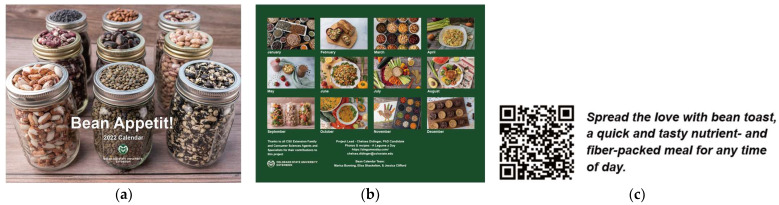
Bean calendar. (**a**) Bean Appetit calendar front cover; (**b**) back cover (the final printed version also contains the logo of the printing company in the bottom right); (**c**) an example of text and the associated QR code that links to the Food Smart Colorado website for the associated recipe post (in this case: a bean toast).

One thousand copies of the Bean Appetit calendar (see Figure 1) were printed and broadly distributed. Although most of the calendars were given to Coloradans due to the nature of the project and its ties to CSU Extension, calendars were sent to people in at least 24 states, 6 countries (USA, Canada, Mexico, South Korea, United Kingdom, Chile), and 4 continents. Some Extension agents gave them away as incentives and prizes to participate in community programs.

#### 3.2.2. Social Media

**Figure 2:**
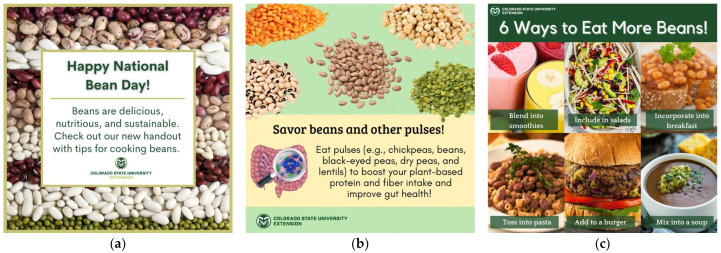
Food Smart Colorado social media bean post images. (**a**) The January 2023 post directed people to the newly created bean handouts that were part of the Extension toolkit; (**b**) This March 2023 post shared nutritional and human health benefits of beans, major motivators for consumption; (**c**) This post from June 2023 was intended to inspire audiences with the culinary versatility of beans, to help address the barrier of unfamiliarity.

Monthly social media posts were published on Food Smart Colorado’s social media platforms, Instagram and Facebook. Figure 2 shows examples of the images, which were also supplemented by captions with helpful information and links to accompanying pages on the Food Smart Colorado website when relevant. For instance, the post shown in Figure 2a read, “Happy National Bean Day!! To celebrate, check out our new handout with tips for cooking dry beans: https://foodsmartcolorado.colostate.edu/cooking-dry-beans/. Beans are a great way to start the new year off right, and then to continue enjoying all year long.” Figure 2c had the caption, “No matter whether you made a new year’s resolution to include more beans this year, or you are a longtime fan of beans, it’s always nice to try new bean dishes. Beans are incredibly versatile and easy to incorporate in any meal of the day—salads, soups, pastas, and more!” Related hashtags were also included.

Other organizations and individuals shared the social media posts, expanding audience reach. As of 13 July 2023, the post with the highest reach (*n* = 669) and engagement (*n* = 69) was from February 2022. This post shared the bean toast recipe from the Bean Appetit calendar, and the caption read, “February is American Heart Month! Did you know that beans are associated with reducing the risk of heart disease? Plus, they are delicious and versatile. Check out this quick and tasty recipe for Bean Toast: https://foodsmartcolorado.colostate.edu/bean-toast/.” The reasons for posting this recipe included: (1.) inspiring people with new ways to use beans; (2.) offering a quick, simple recipe that is more likely to appeal to people because it is easy to make; and (3.) highlighting the association of beans and heart health [4,52] and linking it to the larger narrative of American Heart Month.

#### 3.2.3. Bean Handouts

Two new CSU Extension handouts were developed as part of the Extension Bean Toolkit. “Cooking Dry Beans” (find online: https://foodsmartcolorado.colostate.edu/cooking-dry-beans/) explained how to purchase, store, and cook dry pulses. “Tips for Cooking with Dry Beans and Other Pulses” (find online: https://foodsmartcolorado.colostate.edu/tips-for-cooking-with-dry-beans-and-other-pulses/) offered specific tips to shorten cooking time. Details on the development of this second handout are detailed in our recent publication about consumer-accessible ways to cook dry pulses more quickly [35]. The handouts were distributed as part of the Extension class, as well as being made available online at Food Smart Colorado at the aforementioned links.

#### 3.2.4. Extension Bean-Related Blog Posts

In 2022, the lead author wrote three pulse-related blogs on Live Smart Colorado, a CSU Extension blog run by Family & Consumer Science Extension Agents and Specialists at CSU that publishes weekly. For example, one of the blogs was about how beans can be a secret ingredient in smoothies: https://livesmartcolorado.colostate.edu/try-adding-this-secret-ingredient-to-your-smoothies/. This blog post was one of the most successful Live Smart Colorado posts of the year, ranking number six for top blog posts of 2022. Blogs written earlier in 2022 and in previous years were also evaluated, meaning they had more time to accumulate views throughout the year. Despite being published in June, the smoothie blog still had one of the highest rankings. This suggests that consumers were interested in this topic, which directly relates to both a barrier (i.e., unfamiliarity) and motivating factors (i.e., culinary versatility and boosting nutrition through beans) for pulse intake.

### 3.3. Extension Class

Sociodemographic data was only collected on the pre-survey; thus, data was not collected for all participants in the validation class because not everyone completed the pre-survey. Therefore, demographic data is only shared for the final class, which had more participants and a complete data set of demographics. As this class was conducted through CSU Extension, most participants were from Colorado (83.7%). Of the 69 Coloradoans who completed the survey and chose to list their county, attendees came from 22 of Colorado’s 64 counties. About 88% of participants were female and around 90% White. The largest age group was 60–69 years of age (about 31%), which falls within the average age found for Extension volunteers like Master Gardeners [53]. This was likely because CSU Extension played a key role in helping recruit participants. More detailed participant demographics for the final class are provided in Supplementary Materials File S7.

On the validation class pre-survey, participants were also asked about what they hoped to learn in the class. This was one way to confirm whether the Food Habits Survey and literature review facilitated successful emphasis on topics of consumer interest. Indeed, responses aligned well with class content, grouping into the following categories:

- Cooking and preparation tips (example quotes: “Tips to spend less time cooking dry beans,” “More about beans and ways to cook dry beans instead of canned,” and “More about easiest way to prepare dry beans incl ways to shorten prep time, resources for recipes, resources for buying local”);
- Health information (example quotes: “Health benefits, how to prepare and recipes,” and “Ways to reduce gas in beans, ways to add beans to foods or recipes, frequency of eating that is the best for health”);
- Benefits for the planet (example quote: “Ways that growing beans are beneficial to us and the earth”);
- Diverse ways to use beans/recipes (example quotes: “I cook pintos 95% of the time and would like more diversity,” “New recipes and ideas for preparing pulses,” and “Perhaps some more ways to incorporate dry beans other than the traditional way I fix them, and to broaden which dry beans I have in my diet”); and
- Convenient, tasty ways to eat beans more regularly (example quotes: “Fast cooking tasty recipes,” “I’d also like to have some easy to prepare recipes,” and “Tasty meals and snacks using pulses so I can consume more”).

#### 3.3.1. Changes to Consumption and Bean Preparation Habits

**Figure 3:**
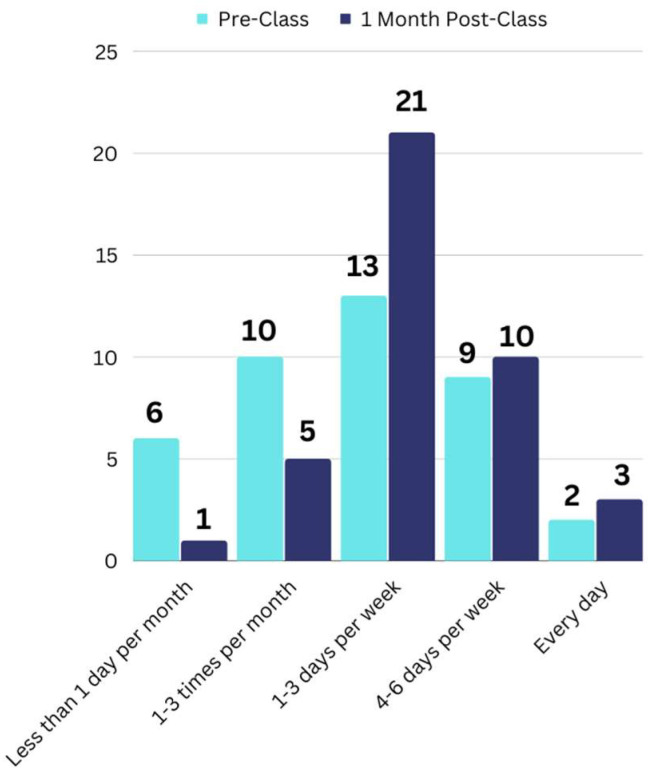
Changes in frequency of pulse consumption after participating in the validation class. “Less than 1 day per month” is shortened in the figure, but the full response option read “several days per year, but less than 1 day per month.” No participants selected “never” as the response for how often they eat pulses. The actual numbers of responses out of *n* = 40 are shown above each column.

The validation class surveyed participants at multiple time points, allowing for follow-up about changes to consumption and preparation behavior. Forty participants filled out the pre-survey and 1-month follow-up surveys, which asked about frequency of consumption and usage of dry versus canned pulses. There was a significant increase in the frequency of pulse intake (*p* = 0.004), as shown in Figure 3. Changes seen on the higher end of pulse consumption were minor, i.e., similar numbers of participants consumed pulses 4–6 days per week or every day both before and 1 month after the class. However, there was a large jump in the number of individuals eating pulses 1–3 days per week, instead of only 1–3 days per month or less than 1 day per month. In addition, 40.4% of respondents indicated that as a result of the class, they “started cooking with dry pulses more often (as opposed to canned).”

Due to only having one time point for the survey, it was not possible to assess short-term changes in behavior for the final class as was done for the validation class. However, participants were asked if they were “more likely to regularly eat more beans and other pulses” as a result of the class. The majority of participants indicated yes (*n* = 63, 73.3%). Part of why this number is not higher is that some participants commented that they already ate high levels of pulses and therefore would not be increasing this further. For example, one individual stated, “I eat quite a few as is.” Similar sentiments were expressed in the validation class survey, such as “I eat dried beans twice a day [lunch + dinner] and this has not changed due to the class.”

#### 3.3.2. Changes to Knowledge and the Importance of Motivators and Barriers

The final class assessed participant knowledge about three different topics, as well as the importance of several motivators and barriers to pulse consumption (*n* = 86 completed the survey, although actual Zoom participant count was about 130). Table 8 shows changes to participant knowledge before and after the class. Significant increases in participant knowledge occurred for all three categories. The greatest improvement was seen for knowledge of the nutrition and health benefits of beans and other pulses.

The importance of motivators and barriers before and after participation in the final class was also evaluated, as shown in Table 9. High numbers for motivators represent strong motivation and high numbers for barriers are strong barriers (i.e., lower numbers indicate that something is not a big barrier). Before the class, the most important nutritional motivator was protein, but after the class, dietary fiber was the most important. Overall, micronutrients (i.e., vitamins and minerals) saw the greatest increase in importance among the nutritional motivators. For the other motivators (i.e., non-nutritional motivators), before the class, taste was ranked as the most important. However, after the class, both human health benefits and sustainability ranked higher than taste. Even though “local” had the lowest of the importance rankings post-class, it increased by the greatest amount. For barriers before the class, long cooking times were the greatest barrier, followed by being unsure how to prepare pulses. People appeared to like the taste of pulses and thus did not report that taste discouraged them. All barriers saw a decrease in importance after the class, meaning they less strongly discouraged people from eating pulses. The greatest decreases in importance were seen for the top two barriers, long cooking time and unsure how to prepare, indicating that the class successfully helped mitigate these barriers.

#### 3.3.3. Themes in the Free Response Data

Recurring themes were found in the data. In the tables below, quotes from the validation and final class have been combined because class content was the same. Additional participant quotes demonstrating these themes are provided in Supplementary Materials File S8. Table 10 groups responses into themes for two separate, but similar, questions: (1.) “What information shared, if any, most motivated you to eat more pulses?”; and (2.) “What did you find most interesting about the class?”

In addition to the themes outlined in Table 10 above, several participants commented that the tips provided for reducing gas helped motivate them to eat more pulses. For example, “that soaking them can reduce flatulence” and “learning what a good source of protein they are, and ways to lessen gassy side effects” were both responses to the survey question about motivation. Moreover, three additional topics were repeatedly mentioned when asked about what they found most interesting in the class. They are listed separately here and not in the table because the same theme was not found for responses to what participants found most motivating.

1. **Clarification about terminology**. Many participants were not previously familiar with the term ‘pulse’ (example quotes: “Definition of Pulses—did not know that term(!!)” and “That after all these years of life, I was not aware of a ‘pulse’”);
2. **Myth busting**. Addressing common bean myths, such as that adding salt always slows down cooking (example quote: “The idea of adding salt—that it doesn’t slow down the cooking of beans”); and
3. **Format**. Participants enjoyed the class format and said that it held their interest (example quotes: “I liked hearing and seeing a little about everything. It kept me focused,” “The topics covered in the class were connected seamlessly to the next topic resulting in an attention grabbing one hour!” and “It was a very cool and informative class, I really enjoyed the pacing of it, the easily digestible info, and cooking tips and recipes!”).

Table 11 highlights the responses to the question that was asked on the post-survey for the validation class and the survey for the final survey, “What new ways, if any, are you looking forward to including pulses in meals?” For the validation class 1-month follow-up survey, participants also responded to the question, “If you have tried a new way(s) to eat pulses since the class, please share what it was and what you thought.” Therefore, the table shows ways in which participants reported having actually acted upon their responses regarding what pulse dishes they were looking forward to trying.

In the surveys, participants also provided information about changes they intended to make to pulse preparation habits and intake. This information is displayed in Table 12. Quotes like “needing something that cooks more quickly” highlight the importance of convenience for consumers, so that pulses can fit into their busy lifestyles.

In the 1-month follow-up survey for the validation class, several individuals commented about having implemented some of the preparation and intake habits mentioned in Table 12. For example, one participant tried cooking beans on the stovetop, as opposed to the slow cooker, and reported, “I had always cooked pintos in the slow cooker and thought they were a little mushy. I cooked them on the stove top and liked them much better!” Another individual stated that they, “Froze cooked beans (made from dried beans) to reheat and eat again.”

## 4. Discussion

### 4.1. Food Habits Survey

#### 4.1.1. Pulse Intake Frequency and Preparation Habits

The majority of participants were from the United States, and 46.6% of respondents chose the option that indicated they consume pulses 1–3 days per week. Although this does not provide an exact number of days per week that pulses were consumed, it falls within the range of frequency of consumption found in other studies. For example, the Global Diet Quality Project shows that 39% of individuals in the United States indicated having eaten pulses the day before they were surveyed [54]. Also, a study by Mitchell and colleagues found that in 2013–2014, approximately 24% of adults ate pulses at least once over the intake period of 2 days [9]. This number is higher than a previous study by Mitchell, which reported that only 7.9% of Americans ate pulses on any given day [55].

Findings about the average frequency of pulse intake by dietary pattern (see Table 2) also align with prior research. Those following vegetarian and vegan diets tend to consume pulses more frequently [12,28,41]. Work by Henn and colleagues revealed a similar trend, with vegetarians and vegans consuming pulses more often, in addition to eating a wider variety of pulses [12]. Henn and team also provided a dietary pattern response option of flexitarian, defining it as “vegetarian with occasionally meat or fish.” In the research herein, flexitarian was not provided as a response option, but instead there was the option of “pescatarian.” In the future, it could be helpful to still include pescatarian, but to separate omnivores into flexitarians with lower meat consumption and those with higher meat consumption.

In a recent survey of university students (*n* = 1433) in the United States aged 18–33, 66% of respondents indicated they soaked pulses before cooking, and 66% prepared them on the stovetop, 17% in a pressure cooker, and 9% in a slow cooker [28]. Of the 882 individuals in this study who were asked about how they cooked dry pulses, 594 indicated that they regularly used the stovetop, or 67% of respondents. Although this number matches that seen by the study among college students, the percentage that indicated using a slow cooker differed, with 350 of the 882 individuals (~40%) indicating regular use of a slow cooker. These differences could be due to how the question was asked, with the current study allowing individuals to select more than one option for how they regularly cook pulses. In addition, the average age of respondents was higher in this study, so they may have better access to kitchen tools, like a slow cooker. Conversely, college students may not own these tools and therefore may rely more heavily on simple cooking practices that only require a pot and the stove.

With regards to preparation of dry pulses, the survey supported that there is confusion over the use of salt. Indeed, the impacts of salt on cooking time are one area of misinformation and myths about dry pulse cooking, as addressed in our recent paper [35]. While some people indicated not using salt due to thinking it prevents pulses from softening (*n* = 137), almost the exact same number (*n* = 139) reported they use salt because it does help with softening dry pulses. Development of resources such as the CSU Extension handouts associated with this research can help mitigate this problem.

#### 4.1.2. Pulse Preference and Form in Which Pulses Are Eaten

When asked about their favorite types of pulses, dry beans ranked as having the highest participant liking, followed by chickpeas. Other studies in North America have found that black beans, lentils, pinto beans, and chickpeas are the most common pulses eaten [28,56,57]. In the current study, cowpeas (also called black-eyed peas) were the least familiar pulse, with nearly 10% of people indicating they had not tried cowpeas before. This highlights the potential to increase consumer exposure to and awareness of how to use this type of pulse.

Previous literature has concluded that unfamiliarity with pulses and how to use them poses a major barrier to higher intake [11,13,41]. Examining the ways in which respondents ate pulses during the last year (see Table 4) helps better understand current consumption habits and provide insights about what to highlight to consumers. For instance, the two least commonly selected response options were “breads, crackers, or pastas made with pulse flour” (*n* = 407, ~43%) and “dessert” (*n* = 192, ~20%). It appears that consumers are not utilizing these ways to include beans in their diets more regularly, potentially due to simply not being aware of these options. Highlighting these options through the toolkit and class could help mitigate unfamiliarity and expand consumer awareness of pulse versatility. This is reflected by the consumer interest expressed in trying these types of pulse dishes after completion of the Extension class.

Dishes like soups, chili, and refried beans were selected by more participants (*n* = 705, 697, and 655, respectively). This aligns with findings from Doma and colleagues, that found that when asked about their favorite recipes, chili and soup were the most commonly reported responses [13]. The manner in which the question was asked in the Food Habits Survey does not allow assessment of frequency of consumption of these dishes. However, when Winham and colleagues asked participants to indicate “eat often,” “have eaten,” and “never eaten” with regards to types of pulse dishes, “chili made with beans” had the highest percentage of “eat often” responses at 35.3% [28].

To address the barrier of unfamiliarity, recipes associated with the bean calendar were created, highlighting the potential to use pulse-based pastas, desserts, and salads. Versatility was covered in the class through emphasizing examples of dishes, the various forms of pulses (e.g., whole, mashed, flour), and ways to stock the pantry and home (e.g., dry, canned, frozen, and pulse-based pastas and other products).

#### 4.1.3. Motivators and Barriers to Pulse Intake

A key purpose of the survey was to determine key motivators and barriers for the potential target audience of the Extension Bean Toolkit resources and class. Of the nutritional attributes of pulses (see Table 5), protein ranked as the most important, followed closely by fiber. A study on factors that influence pulse consumption in Canada also found that both protein and fiber are of key importance, although protein tends to rank above fiber [41]. In a recent study in Poland (*n* = 1027), 72% of participants perceived pulses to be a good source of protein. However, it is notable that this was a more common perception for women than men, with 81% of women agreeing versus only 62% of men [58].

With regards to other (i.e., non-nutritional) motivators, taste was the most important, followed by health benefits (see Table 6). These two response options had the lowest SDs, indicating that there was less of a spread in respondent choices and more agreement about the importance of these aspects. Other studies have also found taste and sensory aspects to be major motivators, along with health benefits [12,13,26,41]. For example, a study on five European countries found that overall health was the major driver of consumption, followed by sensory preferences [12]. Doma and team examined bean consumers (defined as those who consume beans on a daily or weekly basis) and non-consumers (defined as those who consume beans on a monthly basis or never) [13]. They found that the top two motivators were nutritional value and taste and/or texture [13]. Interestingly, the order of importance was reversed for these two groups, although the differences were slight. The authors showed that 93.8% and 92.2% of bean consumers reported that nutritional value and taste were important, respectively. Yet, non-consumers ranked taste as more important (83.6%) than nutritional value (81.2%).

The average ranking of “gluten-free” was 2.46 on a 5-point Likert scale and had the highest SD. This suggests that this attribute was largely unimportant to participants and that the perceived importance varied more widely than other motivators listed. For those not following a gluten-free diet, it is understandable that this attribute may not be important, as gluten-free is more of a niche market [41]. However, there also appears to be a lack of awareness that pulses are naturally gluten-free. For example, even among registered dietitians surveyed in a recent study, about 13% of respondents were unaware that those with celiac disease could eat beans [59].

With regards to barriers to cooking dry pulses, the major barrier was long cooking time (*n* = 449, ~48%). Long cooking time is an established barrier to pulse intake [11,18,35]. The next most common barrier to cooking with dry pulses was simply being unsure of how to cook dry pulses. Overall, this suggests that consumer resources with practical cooking information and tips on how to reduce cooking time could be helpful, and therefore they formed part of the resulting Extension Bean Toolkit.

### 4.2. CSU Extension Bean Toolkit and Class

The goal of the Extension Bean Toolkit was to implement a translational research approach and leverage the Extension network to reach a wider audience with information to encourage increased pulse consumption. The IMB model, findings from the Food Habits Survey, and a review of the literature provided key insights for toolkit development. The design of a toolkit and class differentiate this research from prior studies. Previous research focuses on investigation of motivators and barriers of pulse intake, but not on addressing consumer interests and barrier mitigation. The resources developed as part of the Extension Bean Toolkit have the potential for long-term impact through continued distribution through the Extension network for years to come. Moreover, this translational research can serve as a model for future outreach research through Extension or other organizations promoting healthy behavior change and public health.

A study on Canadian consumers found that, on an aided basis, “not knowing how to cook or prepare pulses” was one of the biggest limitations to consumption [41]. The same study examined consumer attitudes toward pulses and identified five distinct consumer groups. At one end of the spectrum, there were “Informed Champions” who already embrace pulses. “Disinterested Unreachables” were on the other end, making up a group that is unlikely to regularly incorporate pulses in their diets. The other three groups in the middle were determined to be the best segments to target when trying to increase pulse intake:

1. “Unexposed Reachables” have low consumption, largely due to lack of exposure to pulses. Health benefits resonate strongly with this group, and it can be beneficial to teach them a variety of tasty, basic recipes.
2. “Forgetful Proponents” enjoyed pulses but needed a reminder to include them in their diets more frequently. Providing them with a wide variety of delicious ways and recipes in which pulses can be used was suggested. Additionally, it could be beneficial to remind them of the nutrition and health benefits of pulses.
3. “Health Driven Persuadables” find the taste, health, and environmental benefits of pulses appealing. The main barriers to higher consumption are that they do not know how to cook or prepare pulses and do not think about including them in meal planning. The authors recommended quick recipes to align well with the busy lifestyles of this group. Teaching them how to cook pulses while communicating the health benefits of pulses—along with some of the environmental benefits—is the best way to reach this group.

The Extension Bean Toolkit includes a variety of resources that would appeal to these audiences, and to the fact that while unfamiliarity can be a barrier, culinary versatility is a motivator [11,12,13]. For instance, the Bean Appetit calendar contains creative pulse recipes, many of which are designed to be quick and easy. The calendar pages and associated social media posts included food photography, as recommended by the report from Canada, which emphasizes that “Colourful, delicious looking dishes are a must. Visually appealing pictures are key in any communications or messaging targeting the public—they will make people stop and take notice” [41] (p. 3). The report also states that a website address where consumers can find recipes is a critical component of communications. All of the bean calendar pages and the majority of the social media posts were associated with Food Smart Colorado website pages. The Extension handouts and class also detail the cooking and preparation of pulses, which would be especially important for audiences like the Health Driven Persuadables.

Social media can be a low-cost, effective way to broaden nutrition outreach [60]. For the posts in the toolkit, the health benefits of beans were a major focus. This aligns with the recommendation to focus on this and the fact that Food Habits Survey respondents indicated health as one of the main motivators to eat pulses. The environmental benefits were also mentioned, although they were not emphasized in as much detail as the health benefits. Out of the seven non-nutritional motivators assessed in the Food Habits Survey (see Table 6), sustainability/environmental benefits ranked fourth, or in the middle. The report from Canada also recommended that the environment be a secondary focus because preserving the environment is not likely to drive consumers to increase pulse intake, though it is important to many and is good to address [41]. Similarly, women in the previously mentioned survey of people in Polish cities recognized that pulses were environmentally friendly, but this knowledge would not impact their consumption [58].

### 4.3. Extension Class

#### 4.3.1. Changes to Consumption and Bean Preparation Habits

A similar trend in changes to pulse consumption was seen with the Extension class as was with our recent Bean Cuisine citizen science project [19]. This is notable because whereas the Bean Cuisine citizen science work involved interacting with participants over an extended period of time, the Extension class only included a 1-h online class and the accompanying surveys. Overall, the biggest change seen in both this work and the citizen science project was a large transition in the total number of individuals who ate pulses less frequently before participation to eating pulses 1–3 days per week. Yet, results were only significant for the Extension class and not the Bean Cuisine. This could partially be due to the fact that a greater number of class participants originally only ate pulses less than 1 day per month and saw an increase in consumption frequency to at least 1–3 days per week, whereas the baseline consumption frequency of citizen scientists was higher. When asked if they were “more likely to regularly eat more beans and other pulses,” 73.3% of the Extension validation class participants indicated yes. This matches very well with the 71.4% of citizen scientists who marked yes on the same question [19].

The Bean Cuisine participants demonstrated an increase in the usage of dry pulses as opposed to canned [19]. This was also seen in the current research, where 40.4% of respondents indicated that as a result of the class, they “started cooking with dry pulses more often (as opposed to canned).” This suggests that the barriers of long cooking time and not being aware how to cook dry pulses [11,18] were mitigated through participation. When cooking dry pulses in the home, environmental impact also varies with cooking method and the amount cooked. For instance, cooking larger volumes at a time as opposed to a few smaller batches is one way to reduce carbon footprint [61].

Although several studies have found positive perceptions of canned beans [26,27], a recent study by Heer and Winham revealed that some Latinas have negative views of canned beans [57]. Due to the benefits of pulses for both human and environmental well-being, addressing negative perceptions is important because canned pulses represent a practical, convenient way to increase pulse consumption [62]. Dry pulses generally have a slightly lower environmental footprint than canned pulses—ranging from approximately a one- to fourfold difference—although this depends on factors like pulse type [62]. Yet, both canned pulses and dry pulses cooked in the home have a much lower environmental footprint than animal proteins [62]. Thus, it is important to highlight that choosing pulses as a dietary staple—whether canned or dry pulses cooked in the home—is an effective way for consumers to reduce their carbon footprint [62,63]. To help reduce or eliminate negative perceptions of canned pulses, tips for cooking with canned beans can be provided, e.g., drain and rinse canned pulses to reduce sodium [64]. Overall, canned pulses can be more convenient than dry pulses [62], and the goal of making it easier for consumers to increase pulse consumption is more important for environmental benefits than the form (e.g., canned, dry, frozen) in which the pulses are procured. These are topics to address in more detail in future resources or classes.

#### 4.3.2. Changes to Knowledge and the Importance of Motivators and Barriers

Evidence-based information was shared with the public via the Extension Bean Toolkit, and the impacts of participation in this translational research were evaluated for class participants. Levels of knowledge for all assessed pulse-related topics increased after the class. The standard deviation also decreased, meaning the data clustered more tightly and knowledge levels of participants were more similar after the class than before. Knowledge of how to prepare dry pulses saw the smallest increase. This could be due to a higher baseline knowledge than the other categories, as the overall final knowledge score was similar to that for the other two topics.

There were several key differences in changes to knowledge after participation in the final Extension class as opposed to the recent Bean Cuisine citizen science project [19]. First, initial knowledge levels of the three areas assessed were lower for the Extension class participants (scores of 2.96 for nutrition and health benefits, 2.84 for versatility, and 3.11 for how to prepare dry pulses, on a scale of 1 to 5) than for citizen scientists (scores of 3.86, 3.39, and 3.55 for the same topics). This could be due to the nature of citizen science efforts, which tend to attract a group that is already highly interested in the topic and therefore likely has higher baseline knowledge [20,65]. Another potential explanation is that there may be some response-shift bias with the citizen scientists, who saw the survey questions at different time points. In contrast, the final Extension class participants only saw the questions once, when they completed a retrospective pre-/post-assessment. This is reflected in other research, such as leadership trainee self-reported ratings being significantly higher on traditional pre-tests than on retrospective pre-tests [45]. Providing questions in this retrospective format can also help establish a common metric and offer a more accurate measure of subjective growth [44].

Whether individuals participated in the class or as citizen scientists, the final knowledge scores were similar. Knowledge of pulse versatility was 4.39 for the Extension class and 4.38 for the citizen scientists, and how to prepare dry pulses was 4.40 for the class and 4.36 for citizen scientists [19]. This does not assess long-term changes to knowledge, as participation in the citizen science project could have imparted skills and improved self-efficacy regarding pulse dish preparation that may not occur during an online class. Furthermore, gains in knowledge about nutrition and health benefits saw the largest increase for the Extension class but the lowest increase for citizen scientists (an increase of 1.64 points on the 1 to 5 scale versus only 0.43 points) [19]. This might be due to the fact that while the class emphasized the health benefits of pulses, the health benefits were not a main focus of the citizen science work, which primarily highlighted pulse versatility.

For nutritional motivators, prior to participation in the final Extension class, protein was the most important (4.06), followed by fiber (3.99) (see Table 8). Protein and fiber were also the two most important nutritional motivators in the Food Habits Survey (see Table 5). There is a hyper-focus on protein in countries like the United States, despite the fact that most people consume more protein than is required [66] and under-consume dietary components like fiber [67]. In fact, of the four dietary components of public health concern in the United States [68], pulses are rich in two: dietary fiber and potassium. Therefore, pulses can play an important role in reversing the dietary fiber gap (i.e., the dramatic difference between the recommendation and amount of fiber actually consumed) and increasing consumption of these dietary components [14,67]. Improving public awareness of how pulses provide a practical and healthful solution to this problem is critical, in conjunction with practical tips of how to apply this knowledge.

After the class, fiber scored most highly (4.76), followed closely by micronutrients (4.71) and protein (4.70). Micronutrients saw the greatest increase in importance, gaining 1.15 points on a 5-point Likert scale. This could be due to several factors, such as lower baseline importance ranking (3.56) compared to protein and fiber, and less awareness of the micronutrients in pulses compared to other nutrients. For instance, although a majority of older adults correctly identified that beans are high in fiber and low in fat and cholesterol, a much smaller percentage successfully identified that beans are high in iron, potassium, and folate [69]. Even among a group of registered dietitians (RDs), the awareness of micronutrients in beans is more limited than one may expect [59]. For instance, approximately 77% of RDs recognized that beans can increase dietary folate, but 16.5% did not know this, and 6.2% disagreed with this correct statement [59]. Individuals with higher pulse intake have been found to have higher intake of micronutrients like magnesium, potassium, folate, and iron [9], so raising awareness about the micronutrient content of beans is key.

Regarding other motivators, taste (4.01) ranked as the most important prior to the class. This is in agreement with the Food Habits Survey findings and the literature, such as the report from Canada that determined liking the taste of pulses to be the most frequent reason provided for eating them [41]. Taste was followed by affordability (3.76) and health benefits (3.70) (see Table 9). This differed from the Food Habits Survey, where health benefits—not affordability—ranked as second in order of importance. However, after the class, health benefits (4.85) and sustainability (4.63) were rated the most highly. Differences between the order of importance of motivators could also be attributed to question design. Whereas the Food Habits Survey only asked individuals to assess the importance once and there was no intervention, the class asked people to assess pre- and post-class importance in a retrospective manner.

“Local” was the least important other motivator (i.e., non-nutritional motivator) both before and after the class, but it saw the greatest increase in importance. A magnified perceived importance of pulses being local also was observed in the Bean Cuisine project, with 69.6% of participants indicating they were more likely to choose Colorado-grown pulses (the project was based in Colorado and most participants were from Colorado, thus this reflects local). One barrier to local food purchasing is lack of time and/or an inability to prepare local foods [70]. The class helped equip participants with practical ways to overcome this barrier via class content and the handouts with cooking tips that were provided.

Participants rated long cooking times and being unsure of how to prepare pulses as the greatest barriers before taking the class. Interestingly, flatulence—which has been attributed to preventing some people from eating beans [39]—only had an average ranking of 2.67 (2 = very minimally discourages and 3 = minimally discourages). This means flatulence did not represent a major barrier. Being unsure how to prepare pulses saw the greatest change pre- and post-class, with an average ranking of 1.81 after the class (1 = does not discourage and 2 = very minimally discourages). This aligns with the increase in knowledge of pulse versatility seen due to the class.

#### 4.3.3. Themes in the Free Response Data

When asked about what information they found most interesting and motivating in the class, health benefits and nutrition were major themes. Under health benefits, gut health was frequently mentioned, reflecting the current heightened consumer interest in gut health [71]. Regarding nutrition, protein and fiber were mentioned more frequently than micronutrients like potassium. This is likely due to high consumer interest in protein [66], but it also demonstrates the importance of further emphasis about micronutrients in future resources and class content. Culinary versatility was another prevalent theme in the data, which is in agreement with knowledge increases and a reduction in the barrier of lack of awareness of how to use pulses. Several class participants expressed an interest in trying Mayocoba beans, which they heard about for the first time in the class. The Bean Cuisine also resulted in a greater awareness of pulse variety and a liking for Mayocoba beans, with participants making statements like, “My first time having Mayocoba beans; wow, love them!” [19] (p. 11). Another theme revealed was the importance of presenter enthusiasm, which helped provide motivation to eat more beans. In education, enthusiasm is regarded as one of the most essential qualities of an effective teacher [72].

Overall, capitalizing on themes revealed in the free response data about what most intrigues and motivates consumers is one way to better capture public interest in pulses. Comments from class participants show that the tips about how to reduce gas also helped motivate them to eat more pulses (e.g., “learning…ways to lessen gassy side effects” was a response to what motivated someone). This demonstrates the importance of not only emphasizing motivators but also addressing potential barriers when trying to motivate increased pulse intake. In addition, it is important to reach a variety of people, even if they could be considered bean consumers, as shown by the quote, “Beans have always been a part of my diet, but I stopped eating them this past year; however, after the presentation, I have decided to include pulses in my diet once again.”

Class participants also responded to what type of pulse dishes they looked forward to trying (see Table 11). A wide range of pulse dishes emerged, suggesting that participants better recognized the many versatile uses of pulses after the class. Crucially, individuals also mentioned their social circles in their responses, revealing the potential of a positive ripple effect, wherein class participants shared information and recipes with their friends and family and prepared pulse dishes for others. For example, participants expressed that they talked to their families about eating beans more frequently, shared what they learned with many friends, and that they would include pulses in their cooking (e.g., add beans to smoothies when making them with their kids). People even indicated trying recipes immediately after the class (e.g., “I made black bean brownies for the first time right after the talk”).

Responses revealed potential areas to expand upon in future resources. For example, most people who mentioned baking talked about sweet dishes, indicating that savory baking applications could also be highlighted to expand horizons. Although people were intrigued by the idea of beans for breakfast, they seemed to need more quick, tasty ideas on how to accomplish this. One individual commented “I’m still getting used to the texture. It’s a bit heavy.” Thus, suggesting an array of recipes that include pulses but are light in texture could be more appealing. Other comments received agreed with previous research. For example, one individual wrote that they previously had beans “mainly for dinners and in soups” and another that “before the class I only ate them as a side dish or in chili or bean soup”. This directly aligns with findings by Doma and colleagues, which show that beans are most often consumed during the dinner meal and in dishes like soups or chilis [13].

Participants also provided helpful ideas of ways to incorporate beans, expressing that they were “too lazy” to make recipes so instead “just try to add a bit to every lunch and dinner”, for example by sprinkling them on salads. This demonstrates the potential of people to provide bottom-up, practical solutions to increase pulse intake that would resonate with other individuals. One way to capture and disseminate such solutions would be to use the National Weight Control Registry [73] as a model to create a National Bean Registry. We proposed this concept in a recent paper [14]: “The registry would prioritize a bottom-up approach, working with individuals already consuming high levels of pulses to determine what motivates their eating patterns and disseminating information collected from them, such as meal tips” [14] (p. 9).

### 4.4. Limitations and Future Directions

This study has several limitations. As previously explained, the participant groups had a relative lack of sociodemographic diversity, which could limit the generalizability of findings. An additional concern is the recruitment of class participants who are already familiar with and interested in beans. Although this is a consideration, it appears that people with low knowledge of pulses also enrolled (e.g., “Before this class, I truly knew nothing about beans! Learning the difference between beans and pulses, and how to cook with them was great. Everything from learning what to buy, how to store, how to soak, etc. was very helpful.”). Furthermore, it is still important to remind those who are aware of beans to include them in their diet. This is suggested by participant comments that the class encouraged them to begin to include beans in their diet again and the segmentation analysis from the Canadian report that detailed approaches to reach Forgetful Proponents [41].

Another challenge was not using a survey instrument that allowed for more detailed data collection on the amount and types of pulses consumed. The current survey collected information about a range of consumption frequency, not the exact number of days per week/month, how many times per day, the volume consumed, or the timing of consumption (i.e., for what meals of the day). Lack of an assessment tool to evaluate more nuanced information about pulse consumption poses a challenge to understanding consumer behavior, and thus the ability to extract helpful and consumer-accessible tips to eat pulses more regularly [14]. Strides are being made, for instance in the more detailed assessment tool used in a recent study by Henn and colleagues [12]. Gathering information about preparation and consumption habits, motivators, and barriers is important. However, it is also critical to reach the public with the evidence-based information available to help mitigate barriers and emphasize motivators of interest. The current study suggests this can be accomplished through translational research approaches informed by the IMB model, and utilization of the Extension network. Integration of assessment tools that allow for more detailed evaluation of the impacts of adopting a translational approach can provide improved understanding of how to move the needle on pulse intake. They can also provide insights that are informative to implementing successful future public health efforts to encourage healthy behavior change.

Participants directly expressed points of future interest, such as receiving more recipes and learning “how to ‘hide’ the beans in our cooking especially when our spouses are tired of eating beans.” In the future, these points could be addressed. An online class has benefits, like lower cost and the potential to reach a more widely geographically distributed audience. Nevertheless, an in-person class that includes an interactive cooking component could provide hands-on experience and build familiarity and confidence in preparing pulse dishes. This sentiment was expressed by one individual, “It takes energy and time to learn new recipes, and I am afraid that I am not cooking beans in the correct way, and I will waste time and money by implementing the new changes to my diet. In a perfect world, I would love to attend an in-person class about pulses that has a hands-on cooking and tasting component to eliminate my doubts and further increase my confidence in cooking dry beans.” After completion of the online class, two in-person classes were conducted, although without a post-survey, so development is underway.

Additional future directions could include examining impacts on long-term pulse preparation and consumption habits and development of the aforementioned National Bean Registry. Findings from the current research could be integral in the creation of a massive open online course (MOOC) [74,75] to educate a broad audience about pulses and encourage higher intake. Participation in the National Bean Registry and MOOC could be integrated and impacts on behavior change relating to pulses monitored. In the future, researchers could work across sectors to equip policymakers with strategies that successfully encourage increased pulse consumption. Impactful elements and information from the toolkit could be integrated into a public health campaign that promotes pulses. Increasing pulse intake has been shown to associate with dramatic benefits for sustainability and economic savings on healthcare. Switching animal proteins for pulses also has numerous benefits for sustainability [2,5,76]. For example, the single substitution of beans for beef in the United States has been shown to help achieve nearly 50 to 75% of the reductions necessary to meet the country’s 2020 target for greenhouse gas emissions [63]. Also, regular inclusion of pulses in the diet can result in significant health care cost savings due to reductions in chronic disease rates and the associated costs of treatment [77,78]. Such findings further demonstrate the importance of an improved understanding of how to influence the healthy behavior change of adopting pulse-centric diets.

## 5. Conclusions

Beans and other pulses are positioned to simultaneously improve nutritional status, public health, and environmental well-being Yet, they are dramatically under-consumed, largely due to barriers such as unfamiliarity, long cooking times, and intestinal discomfort. One way to encourage increased pulse consumption is adopting a translational approach to ensure research is disseminated to the public in a way that emphasizes motivators and mitigates barriers. The current study conducted a Food Habits Survey in conjunction with a review of the literature to assess points of consumer interest, common motivators, and how to mitigate barriers. The resulting Extension Bean Toolkit provided a variety of resources to reach a wide audience, including website pages, social media posts, and an online class. Participants saw significant gains in knowledge about the benefits of pulses and how to prepare them. Moreover, participation in the class resulted in a reduction in the degree to which barriers discouraged participants and an increase in the importance of all motivators. Participants also reported eating pulses more frequently and an intention to try new pulse dishes.

Other studies on motivators and barriers to pulse intake have focused on investigating the relative importance to participants and on categorizing respondents into different groups. Importantly, this study adopted a translational approach to create a toolkit and class. Then, evidence-based information was actually shared with the public and impacts of participation were measured. Through leveraging the Extension network, a wider audience was reached with critical information that can directly empower them to improve their own health and the health of their communities. Further expanding influence via efforts like a MOOC or the National Bean Registry could have even more widespread benefits. Overall, this study suggests that translational research can play a pivotal role in influencing positive health behaviors, like higher pulse intake. This has potential implications for policy and public health campaigns through organizations like Extension. For instance, it provides insights to guide future translational approaches and efforts designed to promote healthy behavior changes that can benefit human health and sustainability.

## Figures and Tables

**Table 1:** Frequency of pulse intake of Food Habits Survey respondents.

How Often Eat Pulses	Number (Out of *n* = 940)	Percentage of Respondents
Every day	63	6.7
4–6 days per week	229	24.4
1–3 days per week	438	46.6
1–3 times per month	166	17.7
Less than 1 day per month ^1^	40	4.3
Never	4	0.4

^1^ On the survey, this response option was phrased as “several days per year, but less than 1 day per month.”

**Table 2:** Average frequency of pulse intake, by dietary pattern.

Dietary Pattern (*n* Out of 940)	Average ^1^ ± SD	Mode
Vegan (*n* = 69)	5.04 ± 0.74	5 (=4–6 days per week)
Vegetarian (*n* = 84)	4.70 ± 0.77	5 (=4–6 days per week)
Pescatarian (*n* = 106)	4.37 ± 0.88	4 (=1–3 days per week)
Omnivore (*n* = 680)	3.89 ± 0.89	4 (=1–3 days per week)

^1^ A larger number represents more frequent pulse intake, where 1 = never, 2 = several days per year, but less than 1 day per month, 3 = 1–3 days per month, 4 = 1–3 times per week, 5 = 4–6 days per week, and 6 = every day.

**Table 3:** Respondent liking of five main types of pulses.

Pulse Type ^1^	Mode ^2^ Score	Total Likes ^3^ (*n*)	Total Dislikes ^4^ (*n*)	Have Not Tried (*n*)
Dry beans	Strongly like	803	60	4
Chickpeas	Strongly like	766	71	12
Lentils	Strongly like	707	79	18
Dry peas	Somewhat like	612	132	30
Cowpeas	Somewhat like	510	139	89

^1^ Pulses were clarified with the following wording in the survey: chickpeas (also called garbanzo beans); cowpeas (also called black-eyed peas); dry beans (pinto, black, kidney, etc.); dry peas (split peas). ^2^ To calculate the mode, “have not tried” was considered one step further than strongly dislike, as individuals had not been interested enough to even try this type of pulse before. ^3^ This shows the sum of participants who indicated they either strongly or somewhat like this type of pulse. ^4^ This shows the sum of participants who indicated they either strongly or somewhat dislike this type of pulse. Note that the number of neutral responses is not shown in the table.

**Table 4:** How respondents ate pulses within the last year.

Type of Dish	*n* (Out of 940) ^1^
Beans or other pulses with rice or other grains	772
Dips	731
Soups	705
Chili	697
Plain beans or other pulses	658
Refried beans	655
Salads	623
Pastas (pulses mixed in with pasta, not pulse flour pastas)	452
Breads, crackers, or pastas made with pulse flour	407
Desserts	192

^1^ This is the number of individuals who selected yes when asked if they ate this type of pulse dish within the last year.

**Table 5:** Importance of nutritional motivators to Food Habits Survey respondents.

Motivator ^1^	Average ^2^ ± SD	Mode	Total Important ^3^ [*n* (%)]
Protein	4.39 ± 0.81	5	812 (86.8)
Fiber	4.29 ± 0.85	5	809 (86.2)
Micronutrients	4.21 ± 0.87	5	753 (80.6)
Low-fat	3.83 ± 1.09	4	620 (66.2)
Low-calorie	3.65 ± 1.10	4	544 (58.2)

^1^ Participants were asked “How important are the following nutritional aspects of pulses in motivating you to eat them?” ^2^ A higher average score indicates greater importance, with 1 = very unimportant, 2 = somewhat unimportant, 3 = neutral, 4 = somewhat important, and 5 = very important. ^3^ Very important and somewhat important responses were added together, to show the total number of respondents who indicated that this nutritional factor was important in motivating them to eat pulses, with results displayed as *n* (%).

**Table 6:** Importance of other motivators to Food Habits Survey respondents.

Motivator ^1^	Average ^2^ ± SD	Mode	Total Important ^3^ [*n* (%)]
Taste	4.52 ± 0.75	5	851 (90.9)
Health benefits	4.30 ± 0.81	5	790 (84.5)
Affordable	3.99 ± 1.00	5	679 (72.5)
Sustainability	3.98 ± 1.00	5	668 (71.4)
Family & friends ^4^	3.51 ± 1.14	4	509 (54.6)
Tradition/cultural ^5^	3.20 ± 1.15	3	370 (39.5)
Gluten-free	2.46 ± 1.37	1	227 (24.3)

^1^ Participants were asked “How important are the following reasons in motivating you to eat pulses?” ^2^ A higher score indicates greater importance, with 1 = very unimportant, 2 = somewhat unimportant, 3 = neutral, 4 = somewhat important, and 5 = very important. ^3^ Very important and somewhat important responses were added together, to show the total number of respondents who indicated that this nutritional factor was important in motivating them to eat pulses, with results displayed as *n* (%). ^4^ Survey wording was “family and/or friends likes eating beans and other pulses.” ^5^ Survey wording was “part of traditional food choices/cultural reason.”

**Table 7:** Barriers to cooking dry pulses.

Barrier	*n* (Out of 940) ^1^
Long cooking times	449
Unsure how to cook dry	158
Prefer canned	118
Don’t like cooking	105
Don’t have cooking equipment	68
Don’t like pulses	51

^1^ This is the number of individuals who selected yes when asked, “Have any of the following ever prevented you from cooking with dried pulses? Please select all that apply.”

**Table 8:** Pulse knowledge before and after participating in the final Extension class.

Knowledge of Pulse Nutrition and Health Benefits	Pre: *n* (%)	Post: *n* (%)
1 (low)	9 (10.5)	0
2	19 (22.1)	0
3	29 (33.7)	2 (2.3)
4	22 (25.6)	30 (34.9)
5 (high)	6 (7.0)	53 (61.6)
Average score ± SD	2.96 ± 1.10	4.60 ± 0.54
Difference (*p*-value)		1.64 *(<0.001)*
		
**Knowledge of Pulse Versatility ^1^**	**Pre: *n* (%)**	**Post: *n* (%)**
1 (low)	6 (7.0)	0
2	31 (36.0)	1 (1.2)
3	23 (26.7)	9 (10.5)
4	21 (24.4)	31 (36)
5 (high)	4 (4.7)	44 (51.2)
Average score	2.84 ± 1.03	4.39 ± 0.73
Difference (*p*-value)		01.55 *(<0.001)*
		
**Knowledge of How to Prepare Dry Pulses**	**Pre: *n* (%)**	**Post: *n* (%)**
1 (low)	9 (10.5)	0
2	17 (19.8)	0
3	25 (29.1)	8 (9.3)
4	24 (27.9)	35 (40.7)
5 (high)	10 (11.6)	42 (48.8)
Average score	3.11 ± 1.18	4.40 ± 0.66
Difference (*p*-value)		1.29 *(<0.001)*

^1^ The question asked about “knowledge of ways to use beans/pulses in various dishes”.

**Table 9:** Importance of motivators ^1^ and barriers ^2^ before and after participating in the final Extension class.

**Nutritional Motivators**	**Pre: Average ± SD**	**Post: Average ± SD**	**Difference (*p*-Value)**
Protein	4.06 ± 0.97	4.70 ± 0.67	0.64 (<0.001)
Fiber	3.99 ± 0.93	4.76 ± 0.65	0.77 (<0.001)
Micronutrients	3.56 ± 1.06	4.71 ± 0.70	1.15 (<0.001)
Low-fat	3.51 ± 1.13	4.30 ± 1.02	0.79 (<0.001)
Low-calorie	3.47 ± 1.00	4.21 ± 1.03	0.74 (<0.001)
			
**Other Motivators**	**Pre: Average ± SD**	**Post: Average ± SD**	**Difference (*p*-value)**
Taste	4.01 ± 0.91	4.51 ± 0.72	0.50 (<0.001)
Health benefits	3.70 ± 1.01	4.85 ± 0.45	1.15 (<0.001)
Affordable	3.76 ± 0.96	4.42 ± 0.79	0.66 (<0.001)
Sustainability	3.32 ± 1.07	4.63 ± 0.60	1.31 (<0.001)
Local	3.04 ± 1.10	4.41 ± 0.82	1.37 (<0.001)
			
**Barriers**	**Pre: Average ± SD**	**Post: Average ± SD**	**Difference (*p*-value)**
Long cooking times	3.08 ± 1.34	1.97 ± 1.03	−1.11 (<0.001)
Gas/flatulence	2.67 ± 1.41	1.99 ± 1.08	−0.68 (<0.001)
Unsure how to prepare	2.95 ± 1.36	1.81 ± 1.01	−1.14 (<0.001)
Family/friends don’t like	2.24 ± 1.35	1.81 ± 1.10	−0.43 (<0.001)
Don’t like the taste	1.73 ± 1.08	1.60 ± 0.90	−0.13 (0.177)

^1^ Participants ranked how important the listed aspects were in motivating them to eat pulses. A higher score indicates greater importance, with 1 = very unimportant, 2 = somewhat unimportant, 3 = neutral, 4 = somewhat important, and 5 = very important, therefore, a high average score means this aspect was considered highly motivating. ^2^ Participants were asked how important the listed aspects were in discouraging them from eating pulses. A 1 = does not discourage, 2 = very minimally discourages, 3 = minimally discourages, 4 = somewhat discourages, 5 = highly discourages; thus, a higher number means this factor is considered a strong barrier, and a lower number means the factor does not greatly discourage participants from eating pulses.

**Table 10:** What information participants found most motivating and interesting about the Extension class.

Theme	Motivator ^1^—Example Quote	Interesting ^2^—Example Quote
Health Benefits	“Health benefits! I have high cholesterol and see that adding this fiber from pulses in as many ways as possible could be very beneficial for weight management and hopefully cholesterol reduction.” “The health benefits were more than I realized.”	“Significant benefits of consuming beans from the standpoint of both heart and gut health.” “Understanding the health benefits and your gut will adjust to eating more beans.”
Nutrition	“The stats on dietary fiber were quite eye-opening.” “I didn’t appreciate just how much more fiber they have than oats and other grains.” “I learned that pulses offer more nutrients than my usual breakfast of oat bran, so now it’s ‘beans for breakfast!’ for me!”	“The amount of fiber and protein in beans. I knew they were good for you, but I never really knew the exact reason why.” “The entire class was interesting, but the fact about our dietary fiber gap really surprised me. Also that the pulses are so much higher in protein and fiber.”
Environmental Benefits	“The tremendous health benefits for low cost, both monetary and environmental.” “How good they are for the environment as compared to meat.”	“Interesting about drought tolerant which is more and more important.” “Benefits to the soil and environment.”
Affordable	“Cost-very budget friendly.” “They are a cheaper source of protein.”	“That beans are a triple winner: highly nutritious, inexpensive, and easy on our environment.”
Culinary Versatility	“I appreciated the information and ideas of adding beans to a smoothie or making a bean dip. So many fun new ways to incorporate getting them into my diet.” “The creative ways to add pulses to dishes and the beautiful and very appetizing pictures of pulses in dishes.” “Use of pulses for every meal. Going to personally challenge myself to incorporate beans into breakfast and smoothie options.”	“So many different uses for beans and a reminder to use them in salads!” “Aquafaba!!! I had no idea this was a thing or that it can be whipped like a meringue.” “Using bean flour.” “I learned about the Mayocoba beans. I had never heard of them before and saw that they were at Walmart so will see if I can find them.”
Local	“I had no idea that Colorado grew and produced so many beans!” “That there are a wide range of pulses grown locally.”	“I didn’t realize Colorado grew so many beans and they can be purchased locally. I’ll look into those. I also want to try the Mayocoba beans, these are new to me.”
Presenter Enthusiasm	“Honestly Chelsea’s enthusiasm and passion alone was totally motivating to eat more beans!” “Chelsea’s enthusiasm, makes me want to try more beans.”	“The presenter was marvelous! She was very upbeat and engaging...and knowledgeable. I bet she could get anybody to eat beans!” “The enthusiasm of the speaker—seriously, makes me want to buy bean and get cooking!”

^1^ Responses to the question, “What information shared, if any, most motivated you to eat more pulses?” ^2^ Responses to the question, “What did you find most interesting about the class?” Additional participant quotes are provided in Supplementary Materials File S8.

**Table 11:** New ways in which class participants looked forward to trying and/or did try pulses.

Type of Dish	Looking Forward To ^1^—Example Quote	New Dish They Tried ^2^—Example Quote
Smoothies	“My kids love smoothies so we’ll add to smoothies.” “Me and those in my household very open to trying pulses in smoothies!”	“I added beans to a smoothie. It was great! I shared that with others who said they would be interested in trying that as well.”
Pulse Products	“Bean based pasta” “Pasta. Whole wheat pasta is not very good. Just discovered chickpea pasta and will try it.	“Started buying chickpea chips instead of corn chips.” “My husband and I tried chickpea pasta … instead of the traditional white pasta we get. It was really good! Much better than the whole wheat pasta.”
Baking & Desserts	“The idea of making desserts more nutritious has got me inspired.” “I will try to include beans in my diet more and in baking! I made black bean brownies for the first time right after the talk.” “I like the idea of the chickpea flour for baking, too.”	“Black bean brownies. Loved them!” “Black bean chocolate mousse. Delicious, dense and filling.”
Combining with Meat	“Half and half when we would typically use all beef.” “Adding them more to meat dishes.”	No quote received
Breakfast	“Breakfast! Never considered that” “I am really going to try and eat a lot more beans, and incorporate them into more breakfasts and lunches since I just have had them mainly for dinners and in soups.”	“Pulses in breakfast foods was new to me. I’m still getting used to the texture. It’s a bit heavy.”
Adding to Favorite Dishes	“I want to try some of the recipes that were shown and try adding pulses to meals we already eat.” “Adding beans to meals we already make.” “I plan to toss them into whatever I make.”	“Before the class I only ate them as a side dish or in chili or bean soup. I tried making a bean dip and it worked but I’m too lazy for that. Now I just try add a bit to every lunch and dinner. For instance I add them to my salads whether that be a meal salad or a side salad.”
Salads	“More use in salads!” “Adding beans to salads. I love one meal salads!”	“Added canned chickpeas to a salad--very good.”
Dips	“I really like hummus and think that it will be easier to incorporate it into more meals as a start.” “Easy—first thing I’m going to try: Olive-y Bean Dip!!! YUM!”	“I tried the spread with the olives, and it was delicious!” “Made hummus with black beans.”
Aquafaba	“Using cooking water for egg whites,” “I want to try whipping bean broth. Will use bean broth in making soup.”	“Used the bean cooking water in a smoothie.”
With Other Grains & Carbohydrates	“Adding them to pasta” “Combing them with mashed potatoes”	“I have put in with my oatmeal!”
New Pulse Varieties	“Try the Mayocoba beans. Never heard of them before.” “Honestly I bought a bunch of beans and am looking forward to trying them all in different ways!”	“I tried northern beans for the first time! Loved them.”

^1^ Responses to the question, “What new ways, if any, are you looking forward to including pulses in meals?” ^2^ For those who participated in the 1-month follow-up survey for the validation class, responses to the question, “If you have tried a new way(s) to eat pulses since the class, please share what it was and what you thought.” The responses do not line up, i.e., responses of what participants actually tried have not been matched to what they indicated they are interested in trying, as these responses came from both the validation and final class and multiple surveys. Additional participant quotes are provided in Supplementary Materials File S8.

**Table 12:** Intended changes to pulse intake and preparation habits.

Theme	Example Quote
Intake	“I obviously need to eat way more and make them a regular part of my diet.” “I would like to start the journey towards 2 c. of pulses each day.” “Found the presentation very informative and am definitely incorporating more pulses into my diet.”
Use Variety	“Look into other options when needing something that cooks more quickly (lentils instead of black beans).” “I had never thought of adding white beans to potatoes, macaroni and kidney beans, bean toast, black beans to a chocolate smoothie, or to egg scrambles or breakfast burrito. My mindset before had been just have soups with beans, add beans to taco salads or as a side dish. So many more choices now so thank you!”
Cooking Dry Pulses	“I’ve never cooked dry beans so I’m very excited to try.” “Using more of dry pulses”
Soaking	“Adding salt to soaking!” “Will soak beans to reduce flatulence.” “I have never tried quick soak method shared in the class today so will give it a try also.”
Cooking Method	“The slow cooker seems like a good option to try.” “I will try other cooking methods.”
Freezing	“Can cook enough to freeze and have on hand quickly.” “I also like the idea of saving time by cooking lots and freezing it.”

## Data Availability

Data are contained within the article or Supplementary Materials.

## Supplementary Materials

The following supporting information can be downloaded at: https://www.mdpi.com/article/10.3390/nu15194121/s1, Supplementary Materials File S1: Food Habits Survey; Supplementary Materials File S2: Extension validation class pre-survey; Supplementary Materials File S3: Extension validation class post-survey; Supplementary Materials File S4: Extension validation class 1-month follow-up survey; Supplementary Materials File S5: Extension final class survey; Supplementary Materials File S6: Food Habits Survey demographics; Supplementary Materials File S7: Final Extension class participant demographics; Supplementary Materials File S8: Additional class participant quotes.
