# Supplementary material for: A Translational Approach to Increase Pulse Intake and Promote Public Health through Developing an Extension Bean Toolkit
Source: Nutrients. 2023 Sep 24;15(19):4121. doi: 10.3390/nu15194121 (PMC10574132; doi:10.3390/nu15194121)
Supplement: Supplementary file 1 [file nutrients-15-04121-s001.zip › Supplementary Materials File S1. Final Food Habits Survey.pdf]

Introduction

Please consider participating in this Food Habits Survey! This online survey is part of a PhD research project being conducted in the Food Science and Human Nutrition Department of Colorado State University to identify preferences, challenges, and concerns regarding cooking and eating beans and other pulses (e.g., chickpeas, lentils). **Even if you rarely eat beans, your responses will provide key insights.** Survey findings will inform development of effective and engaging educational materials, a citizen science project, and outreach by Colorado State University Extension to address consumer interests and needs.

At the end of the survey, please provide your email if you would like to be:
- Entered into the raffle to win one of several $20 Amazon gift cards;
- Contacted about survey findings; and/or
- Emailed when Extension resources about pulses are created.

This survey should take about 10-15 minutes to complete and there are no known risks to participants. Your participation is voluntary, and you may skip any question you choose not to answer. You must be 18 or older to participate. Researchers will keep all information confidential. If you have questions, please contact PhD Candidate Chelsea Didinger, at Chelsea.Didinger@colostate.edu, or Dr. Marisa Bunning, Extension Specialist and Professor, at Marisa.Bunning@colostate.edu. If you have any questions about your rights as a volunteer in this research, contact the CSU IRB at: RICRO_IRB@mail.colostate.edu; 970-491-1553.

Thank you for your valuable time and input – your contribution makes this research possible!

- ○ YES, I voluntarily agree to participate in this research.
- ○ NO, please exit me from this survey.

Cooking and Eating Habits

Thank you for agreeing to participate in this survey. First, we would like to ask about your cooking and eating habits.

Which of the following **best** describes your dietary pattern?

- ○ Omnivore (you include meat, like chicken, fish, beef, and/or pork)
- ○ Pescatarian (eat eggs, dairy, fish and other seafood, but no chicken, beef, pork, or other animals)
- ○ Vegetarian (no meat, but eat foods such as eggs and dairy)
- ○ Vegan (no animal products, including eggs, dairy, and honey)

How many days a week do you eat the following meals? For example, if you skip lunch 2 times a week, move the lunch slider to 5.

	0	1	2	3	4	5	6	7	
Breakfast									☐
Lunch									☐
Dinner									☐

Approximately how often do you eat beans or other pulses? Pulses include chickpeas, lentils, and dry peas like split peas but do NOT include soybeans, peanuts, snap peas, etc. See the graphic below for a more detailed definition of pulses.

- ○ Every day
- ○ 4-6 days per week
- ○ 1-3 days per week
- ○ 1-3 days per month
- ○ Several days per year, but less than 1 day per month
- ○ Never

Pulses are a type of legume that include dry beans like black beans, pinto beans, and kidney beans. Chickpeas, cowpeas (i.e. black-eyed peas), dry peas, and lentils are also pulses. Soybeans and fresh green vegetables such as snap beans and snap peas are NOT considered pulses.

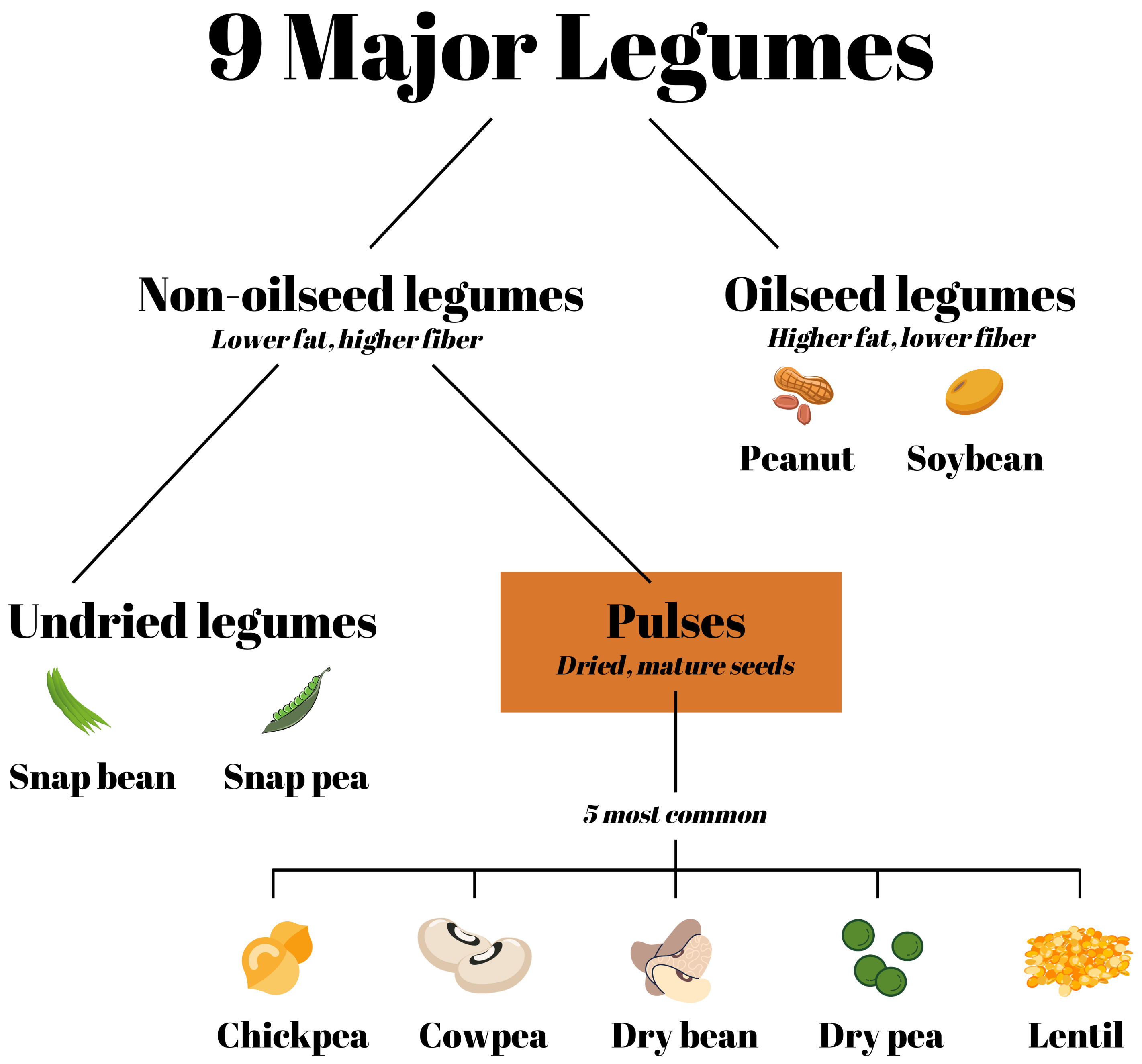

Please indicate how much you like or dislike the following pulses. If you have not tried one of the types of pulses shown below, please select N/A for that type. Note that although pulses like 'dry beans' and 'dry peas' have the word 'dry' in their name, we are referring to cooked pulses.

	Strongly **like**	Somewhat **like**	Neither like nor dislike (neutral)	Somewhat **dislike**	Strongly **dislike**	N/A (have not tried)
Chickpeas (also called garbanzo beans)	○	○	○	○	○	○
Cowpeas (also called black-eyed peas)	○	○	○	○	○	○
Dry beans (pinto, black, kidney, etc.)	○	○	○	○	○	○
Dry peas (split peas)	○	○	○	○	○	○
Lentils	○	○	○	○	○	○

What type of **dry beans** (pinto, kidney, black) do you eat the most? Please only choose **one** DRY BEAN (do not include chickpeas, cowpeas, lentils, dry peas).

____

Please check all the type(s) of pulse dishes you ate in the last year, either in whole form or in mixed dishes. You may select more than one.

- ☐ Beans or other pulses with rice or other grains
- ☐ Breads, crackers, or pastas made with pulse flours (edamame pasta is from soybeans and would not count)
- ☐ Chili
- ☐ Desserts
- ☐ Dips (hummus, layered bean dip, etc.)
- ☐ Pastas (pulses mixed in with pasta, do not include pastas made from pulse flours)
- ☐ Plain beans or other pulses
- ☐ Refried beans
- ☐ Salads
- ☐ Soups
- ☐ Other

____

Approximately how often do you cook with **CANNED** beans or other pulses (including chickpeas, lentils, and dry peas like split peas)?

- Every day
- 4-6 days per week
- 1-3 days per week
- 1-3 days per month
- Several days per year, but less than 1 day per month
- Never

Approximately how often do you cook **DRY** (not canned) beans or other pulses (including chickpeas, lentils, and dry peas like split peas)?

- Every day
- 4-6 days per week
- 1-3 days per week
- 1-3 days per month
- Several days per year, but less than 1 day per month
- Never

Do you have a favorite bean recipe(s) you would like to share? Please consider providing the name of the dish and/or ingredients from 1 or 2 of your favorite, 'go-to' recipes. This will help us compile and design recipe resources.

Block 2

Now, we would like to ask about how you prepare beans and other pulses in the home.

Have you cooked with **DRY** (not canned) beans and other pulses **at least once**?

- Yes
- No

How would you rate your current level of success cooking DRY (not canned) pulses?

- My attempts at cooking pulses are **unsuccessful**.
- My attempts at cooking pulses are **somewhat unsuccessful**.
- My attempts at cooking pulses are **neither unsuccessful nor successful**.
- My attempts at cooking pulses are **somewhat successful**.
- My attempts at cooking pulses are **successful**.

Which cooking method(s) do you regularly use when preparing beans? You can select more than one.

- [ ] Electric pressure cooker (like an Instant Pot)
- [ ] Traditional pressure cooker
- [ ] Slow cooker (such as a Crockpot)
- [ ] Stovetop
- [ ] Oven

How likely are you to soak dry beans and other pulses before cooking?

- Never
- Very rarely (1-20% of the time)
- Somewhat rarely (21-40% of the time)
- Sometimes (41-60% of the time)
- Somewhat often (61-80% of the time)
- Very often (81-100% of the time)

Please briefly explain how you soak pulses.

What type of water do you normally use to soak?

- Bottled
- Filtered
- Tap
- Well

Do you regularly add any of the following to the soaking water? You may check more than

one.

- ☐ Baking soda
- ☐ Salt
- ☐ Other
- ☐ Nothing except water

At what temperature do you normally soak pulses?

- ○ In the refrigerator
- ○ Room temperature

Do you cook the pulses in this soaking water or discard soaking water and use fresh water for cooking?

- ○ Cook in the soaking water
- ○ Discard the soaking water and cook in fresh water

If applicable, when do you choose not to soak and instead cook dry pulses immediately?

Have you ever used the ‘quick soak’ method (boil for about 3 minutes and then let stand for 1 hour before cooking, instead of a longer soak)?

- ○ Yes
- ○ No

Was your experience with the quick soak method favorable or unfavorable, and why?

Is there anything else you would like to share about how you soak and/or cook pulses?

- ○ No
- ○ Yes

Soaking and cooking methods may or may not include salt. When cooking **DRY** pulses, which of the following choices influence your decision to use or not use salt? You can select multiple options.

- ☐ I want to use salt for flavor
- ☐ I think salt helps pulses soften
- ☐ I think salt prevents pulses from softening
- ☐ I want to reduce salt in food for myself and/or my household
- ☐ Following recipe instructions
- ☐ Other
- ☐ I do not use salt in cooking nor soaking

Have any of the following ever prevented you from cooking with dried pulses? Please select all that apply.

- ☐ Do not like pulses
- ☐ Do not like cooking
- ☐ Long cooking times
- ☐ Unsure how to cook pulses
- ☐ Do not have necessary cooking equipment
- ☐ Prefer canned pulses

☐ Other

☐ None of the above

Block 4

Now, we would like to ask about what motivates or discourages you from eating beans and other pulses.

How important are the following nutritional aspects of pulses in **motivating** you to eat them?

	Very **important**	Somewhat **important**	Neither important nor unimportant (neutral)	Somewhat **unimportant**	Very **unimportant**
High fiber	○	○	○	○	○
High protein	○	○	○	○	○
Low fat	○	○	○	○	○
Low calories	○	○	○	○	○
Rich in some vitamins and minerals such as potassium and iron	○	○	○	○	○

How important are the following reasons in **motivating** you to eat pulses?

	Very **important**	Somewhat **important**	Neither important nor unimportant (neutral)	Somewhat **unimportant**	Very **unimportant**
Like the taste	○	○	○	○	○
Human health benefits (may promote gut health and reduce risk for chronic diseases)	○	○	○	○	○
Environmental benefits/sustainability	○	○	○	○	○
Affordable	○	○	○	○	○

	Very **important**	Somewhat **important**	Neither important nor unimportant (neutral)	Somewhat **unimportant**	Very **unimportant**
Part of traditional food choices/cultural reasons	○	○	○	○	○
Family and/or friends likes eating beans and other pulses	○	○	○	○	○
Gluten-free	○	○	○	○	○

If applicable, what other factors not listed above **motivate** you to eat pulses?

____

How important are the following in **discouraging** you from eating pulses? 'Important' reflects a factor that discourages you. 'Unimportant' represents a factor that does not discourage you.

	Very **important**	Somewhat **important**	Neither important nor unimportant (neutral)	Somewhat **unimportant**	Very **unimportant**
Gas/flatulence	○	○	○	○	○
Unsure how to prepare meals and/or snacks with pulses	○	○	○	○	○
Family and/or friends dislike eating beans and other pulses	○	○	○	○	○
Long cooking times	○	○	○	○	○
Dislike the taste	○	○	○	○	○

If applicable, what other reason(s) not listed **discourages** you from eating pulses?

____

Block 3

Almost done! Thank you for your time and making this research possible.

We would like to ask about what resources you currently use regarding pulses, as well as ones you would like to see developed. This will help ensure the outreach resources we design benefit consumers. At the end of the survey, you can indicate if you would like us to send you links to these resources once they are completed.

Please list one - or a few - of your favorite cookbooks, social media platforms, blogs, or websites you regularly refer to for cooking with pulses.

Which of the following types of resources would you be interested in related to pulses?

	Very **interested**	Somewhat **interested**	Neither interested nor uninterested (neutral)	Somewhat **uninterested**	Very **uninterested**
Handouts (printed)	○	○	○	○	○
Handouts (electronic)	○	○	○	○	○
Videos	○	○	○	○	○
Classes with information on pulses and an interactive cooking component	○	○	○	○	○

If taking a class about pulses, would you prefer online, in-person, or no preference?

- ○ Online
- ○ In-person
- ○ No preference

How interested would you be in the following topics?

	Very **interested**	Somewhat **interested**	Neither interested nor uninterested (neutral)	Somewhat **uninterested**	Very **uninterested**
Tips to include more beans and other pulses in your dietary pattern	○	○	○	○	○

	Very **interested**	Somewhat **interested**	Neither interested nor uninterested (neutral)	Somewhat **uninterested**	Very **uninterested**
Nutritional information about pulses	○	○	○	○	○
How elevation affects cooking pulses	○	○	○	○	○
'Myth busters' to address common misconceptions about pulses	○	○	○	○	○
Information on whether pulses really cause flatulence and/or ways to minimize flatulence	○	○	○	○	○
Social media posts (Instagram, Facebook, Twitter, etc.)	○	○	○	○	○

If applicable, please list any other topics of interest related to pulses.

Block 5

Last, please answer a few questions about yourself to help us better understand our audience and make sure we can best develop educational materials to meet your needs and interests.

For the purpose of gaining information of where people are cooking, please provide the following. If you would prefer not to answer, leave the section blank.

Country

State

City

Do you identify as Hispanic?

- ○ Hispanic
- ○ Non-Hispanic

○ Prefer not to answer

With which ethnicity do you most identify? You can choose multiple options.

☐ Asian
☐ Black
☐ Native American
☐ White/Caucasian
☐ Other
☐ Prefer not to answer

Gender

○ Female
○ Male
○ Other
○ Prefer not to say

Age

○ 18-20
○ 21-29
○ 30-39
○ 40-49
○ 50-59
○ 60-69
○ 70-79
○ 80+
○ Prefer not to say

Highest education level

- ○ Some high school, no diploma
- ○ High school graduate or GED
- ○ Some college credit, no degree
- ○ Trade/technical/vocational training
- ○ Associate degree
- ○ Bachelor’s degree, completed or currently enrolled
- ○ Master’s degree, completed or currently enrolled
- ○ Professional degree
- ○ Doctorate degree, completed or currently enrolled
- ○ Other
- ○ Prefer not to say

If you received a higher education degree (Associate or above), in what field(s) did you receive your degree(s)? If you would prefer not to answer, leave the response blank.

Household income level (United States dollar)

- ○ Less than $25,000
- ○ $25,000 - $49,999.99
- ○ $50,000 - $74,999.99
- ○ $75,000 - $99,999.99
- ○ $100,000 or greater
- ○ Prefer not to say

Block 6

Thank you for taking the time to fill out this survey!

Check the box(es) if you would like to be entered in the raffle and/or receive updates. If you do not check any boxes, you will not be entered in the raffle and we will not follow up with survey findings or resources. Your decision to receive/not receive findings and resources does not impact your chance to be entered in the raffle - every survey participant who selects 'I would like to be entered into the raffle for a gift card ' is eligible.

- ☐ I would like to be entered into the raffle for a gift card.
- ☐ I would like to be contacted about survey findings.
- ☐ I would like to be contacted when Extension resources about pulses are created.

Please provide your email.

____

Powered by Qualtrics
