# Supplementary material for: A Translational Approach to Increase Pulse Intake and Promote Public Health through Developing an Extension Bean Toolkit
Source: Nutrients. 2023 Sep 24;15(19):4121. doi: 10.3390/nu15194121 (PMC10574132; doi:10.3390/nu15194121)
Supplement: Supplementary file 1 [file nutrients-15-04121-s001.zip › Supplementary Materials File S2. Extension validation class - Pre-survey.pdf]

Default Question Block

Please take this pre-survey BEFORE participating in the Colorado State University Extension pilot for the Beans: Good for You, Good for the Planet class. This FREE online class is part of a PhD research project being conducted in the Horticulture and Landscape Architecture and Food Science and Human Nutrition Departments of Colorado State University to address topics a recent survey indicated are of interest, such as simple ways to regularly enjoy more beans, dry bean cooking tips, and the many health benefits of beans.

This survey should take about 5 minutes to complete. Your participation is voluntary, and you may skip any question you choose not to answer. You must be 18 or older to participate. Researchers will keep all information confidential. If you have questions, please contact PhD Candidate Chelsea Didinger, at Chelsea.Didinger@colostate.edu, or Dr. Marisa Bunning, Extension Specialist and Professor, at Marisa.Bunning@colostate.edu. If you have any questions about your rights as a volunteer in this research, contact the CSU IRB at: RICRO_IRB@mail.colostate.edu; 970-491-1553.

COLORADO STATE UNIVERSITY
EXTENSION

Your input is critical and appreciated, as this class and the associated surveys are important to the research project and will allow us to improve upon the class and continue to deliver it into the future. As a thank you for participating, the first 100 people who complete ALL 3 brief surveys (this pre-survey, the post-survey after the class, and the 1-month follow-up survey) will receive a $10 Amazon gift card.

Thank you for your valuable time and input – your contribution makes this research possible!

- ○ YES, I voluntarily agree to participate in this research.
- ○ NO, please exit me from this survey.

Block 1

Thank you for agreeing to participate in this survey. First, we would like to ask about your cooking and eating habits. If you participated in our Food Habits Survey, some of these questions may look familiar.

Which of the following **best** describes your dietary pattern?

- ○ Omnivore (you include meat, like chicken, fish, beef, and/or pork)
- ○ Pescatarian (eat eggs, dairy, fish and other seafood, but no chicken, beef, pork, or other animals)
- ○ Vegetarian (no meat, but eat foods such as eggs and dairy)
- ○ Vegan (no animal products, including eggs, dairy, and honey)

Approximately how often do you eat beans or other pulses? Pulses include chickpeas, lentils, and dry peas like split peas but do NOT include soybeans, peanuts, snap peas, etc. See the graphic below for a more detailed definition of pulses.

- ○ Every day
- ○ 4-6 days per week
- ○ 1-3 days per week
- ○ 1-3 days per month
- ○ Several days per year, but less than 1 day per month
- ○ Never

Pulses are a type of legume that include dry beans like black beans, pinto beans, and kidney beans. Chickpeas, cowpeas (i.e. black-eyed peas), dry peas, and lentils are also pulses. Soybeans and fresh green vegetables such as snap beans and snap peas are NOT considered pulses.

9 Major Legumes

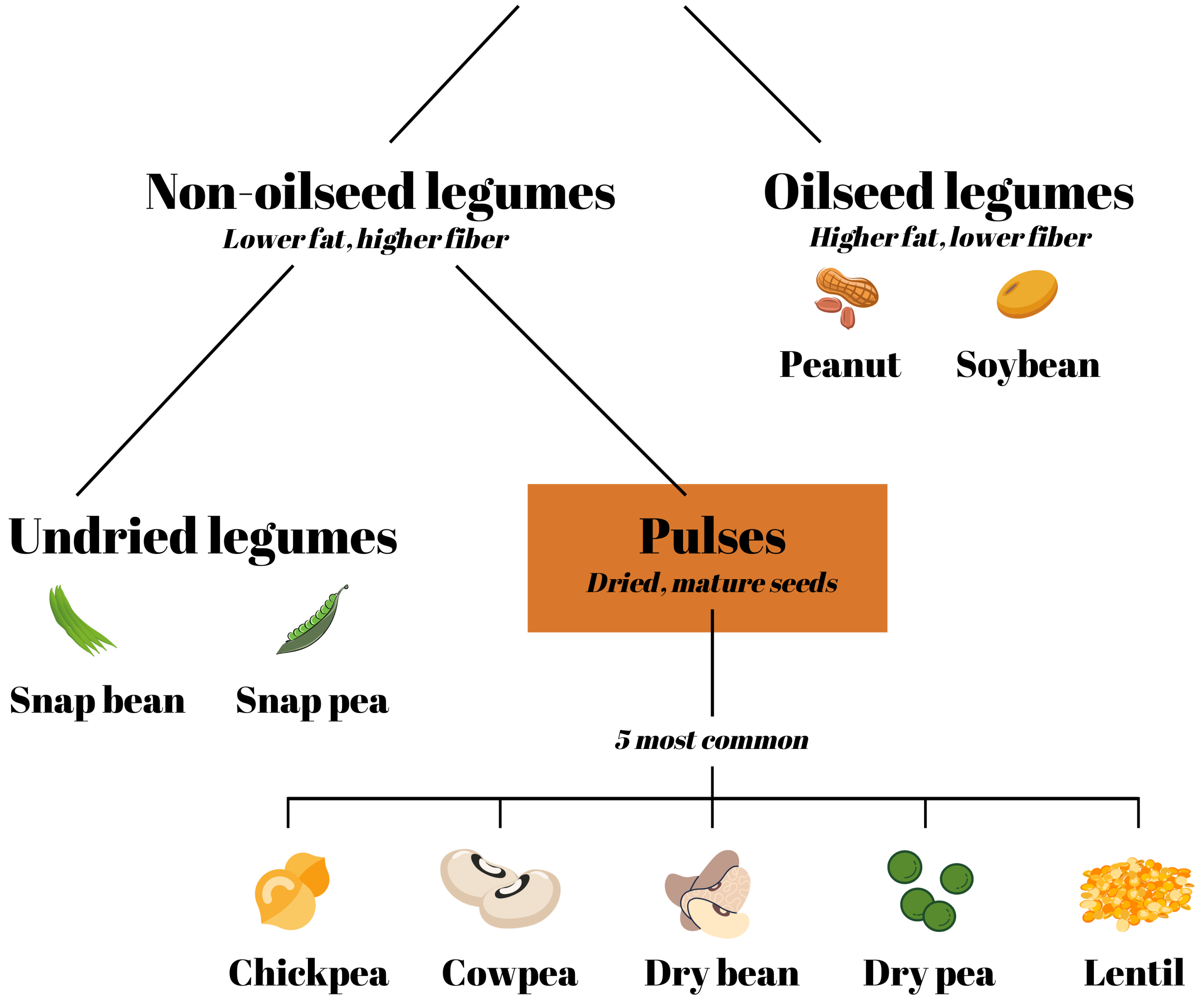

On a scale of 1 (low) to 5 (high), how would you rate your current (**before** taking the class) knowledge of the following?

	1 (low)	2	3	4	5 (high)
Knowledge of bean/pulse nutrition and health benefits	○	○	○	○	○
Knowledge of ways to use beans/pulses in various dishes	○	○	○	○	○

	1 (low)	2	3	4	5 (high)
Knowledge of how to prepare dry beans/pulses	○	○	○	○	○

Which do you more frequently cook with, canned pulses or dry pulses you cook yourself?

- ○ Canned
- ○ Dry
- ○ Approximately 50-50

How often do you currently purchase and eat **Colorado-grown** beans and other pulses?

- ○ Never
- ○ Very rarely (1-20% of the time)
- ○ Somewhat rarely (21-40% of the time)
- ○ Sometimes (41-60% of the time)
- ○ Somewhat often (61-80% of the time)
- ○ Very often (81-100% of the time)
- ○ Unsure if the beans I purchase are from Colorado or not

Approximately how often do you cook with **CANNED** beans or other pulses (including chickpeas, lentils, black-eyed peas, and split peas)?

- ○ Every day
- ○ 4-6 days per week
- ○ 1-3 days per week
- ○ 1-3 days per month
- ○ Several days per year, but less than 1 day per month
- ○ Never

Have you cooked with **DRY** (not canned) beans and other pulses **at least once**?

- ○ Yes
- ○ No

Approximately how often do you cook using **DRY** (not canned) beans or other pulses (including chickpeas, lentils, and dry peas like split peas) cooked in the home?

- ○ Every day
- ○ 4-6 days per week
- ○ 1-3 days per week
- ○ 1-3 days per month
- ○ Several days per year, but less than 1 day per month
- ○ Never

Which cooking method(s) do you regularly use when preparing beans? You can select more than one.

- ☐ Electric pressure cooker (like an Instant Pot)
- ☐ Traditional pressure cooker
- ☐ Slow cooker (such as a Crockpot)
- ☐ Stovetop
- ☐ Oven

How likely are you to soak dry beans and other pulses before cooking?

- ○ Never
- ○ Very rarely (1-20% of the time)
- ○ Somewhat rarely (21-40% of the time)
- ○ Sometimes (41-60% of the time)
- ○ Somewhat often (61-80% of the time)
- ○ Very often (81-100% of the time)

Do you regularly add any of the following to the soaking water? You may check more than one.

- ☐ Baking soda
- ☐ Salt

☐ Other

☐ Nothing except water

Now, we would like to ask about factors that influence how often you eat beans and other pulses.

Have any of the following ever prevented you from cooking with **dry** pulses? Please select all that apply.

☐ Do not like pulses
☐ Do not like cooking
☐ Long cooking times
☐ Unsure how to cook dry pulses
☐ Do not have necessary cooking equipment
☐ Prefer canned pulses
☐ Other

☐ None of the above

How important are the following nutritional aspects of pulses in **motivating** you to eat them?

	Very **important**	Somewhat **important**	Neither important nor unimportant (neutral)	Somewhat **unimportant**	Very **unimportant**
High fiber	○	○	○	○	○
High protein	○	○	○	○	○
Low fat	○	○	○	○	○
Low calories	○	○	○	○	○

	Very **important**	Somewhat **important**	Neither important nor unimportant (neutral)	Somewhat **unimportant**	Very **unimportant**
Rich in some vitamins and minerals such as potassium and iron	○	○	○	○	○

How important are the following reasons in **motivating** you to eat pulses?

	Very **important**	Somewhat **important**	Neither important nor unimportant (neutral)	Somewhat **unimportant**	Very **unimportant**
Human health benefits (may promote gut health and reduce risk for chronic diseases, etc.)	○	○	○	○	○
Environmental benefits/sustainability	○	○	○	○	○
Cost/affordability	○	○	○	○	○
Taste	○	○	○	○	○
Local	○	○	○	○	○

How important are the following in **discouraging** you from eating pulses? 'Important' reflects a factor that discourages you. 'Unimportant' represents a factor that does not discourage you.

	Very **important**	Somewhat **important**	Neither important nor unimportant (neutral)	Somewhat **unimportant**	Very **unimportant**
Gas/flatulence	○	○	○	○	○
Unsure how to prepare meals and/or snacks with pulses	○	○	○	○	○
Family and/or friends dislike eating beans and other pulses	○	○	○	○	○
Long cooking times	○	○	○	○	○
Dislike the taste	○	○	○	○	○

Last, please answer a few questions about yourself to help us better understand who we are serving with Colorado State University Extension programs.

In which Colorado county do you live? If you do not currently live in Colorado, please write 'N/A.'

Gender

- ○ Female
- ○ Male
- ○ Other
- ○ Prefer not to say

Do you identify as Hispanic?

- ○ Hispanic
- ○ Non-Hispanic
- ○ Prefer not to answer

With which ethnicity do you most identify? You can choose multiple options.

- ☐ Asian
- ☐ Black
- ☐ Native American
- ☐ White/Caucasian
- ☐ Other
- ☐ Prefer not to answer

Age

- ○ 18-20

- 21-29
- 30-39
- 40-49
- 50-59
- 60-69
- 70-79
- 80+
- Prefer not to say

Lastly, please tell us what you hope to learn during the Extension class, Beans: Good for You, Good for the Planet.

Thank you for taking the time to fill out this pre-survey!

To match your responses at the three time points, **please provide your email address**. Your responses will not be associated with your name or email - this is simply to help us match survey responses and know to whom we should send a thank you $10 Amazon gift card (available to the first 100 participants who complete all 3 surveys: this pre-survey, the post-survey, and the 1-month follow-up survey).
