# Supplementary material for: A Translational Approach to Increase Pulse Intake and Promote Public Health through Developing an Extension Bean Toolkit
Source: Nutrients. 2023 Sep 24;15(19):4121. doi: 10.3390/nu15194121 (PMC10574132; doi:10.3390/nu15194121)
Supplement: Supplementary file 1 [file nutrients-15-04121-s001.zip › Supplementary Materials File S3. Extension validation class - Post-survey.pdf]

Default Question Block

Please take this post-survey AFTER participating in the Colorado State University Extension pilot for the Beans: Good for You, Good for the Planet class. This online class is part of a PhD research project being conducted in the Horticulture and Landscape Architecture and Food Science and Human Nutrition Departments of Colorado State University to address topics a recent survey indicated are of interest, such as simple ways to regularly enjoy more beans, dry bean cooking tips, and the many health benefits of beans.

This survey should take about 5 minutes to complete. Your participation is voluntary, and you may skip any question you choose not to answer. You must be 18 or older to participate. Researchers will keep all information confidential. If you have questions, please contact PhD Candidate Chelsea Didinger, at Chelsea.Didinger@colostate.edu, or Dr. Marisa Bunning, Extension Specialist and Professor, at Marisa.Bunning@colostate.edu. If you have any questions about your rights as a volunteer in this research, contact the CSU IRB at: RICRO_IRB@mail.colostate.edu; 970-491-1553.

COLORADO STATE UNIVERSITY
EXTENSION

Your input is critical and appreciated, as this class and the associated surveys are important to the research project and will allow us to improve upon the class and continue to deliver it into the future. As a thank you for participating, the first 100 people who complete ALL 3 brief surveys (the pre-survey, this post-survey after the class, and the 1-month follow-up survey) will receive a $10 Amazon gift card.

Thank you for your valuable time and input – your contribution makes this research possible!

- ○ YES, I voluntarily agree to participate in this research.
- ○ NO, please exit me from this survey.

Block 1

Thank you for agreeing to participate in this survey. We would like to ask you to share about your experience and takeaways from the class.

Did you learn something new?

- ○ Yes
- ○ No

As a reminder, **pulses** are a type of legume that include dry beans like black beans, pinto beans, and kidney beans. Chickpeas, cowpeas (i.e. black-eyed peas), dry peas, and lentils are also pulses. Soybeans and fresh green vegetables such as snap beans and snap peas are NOT considered pulses.

9 Major Legumes

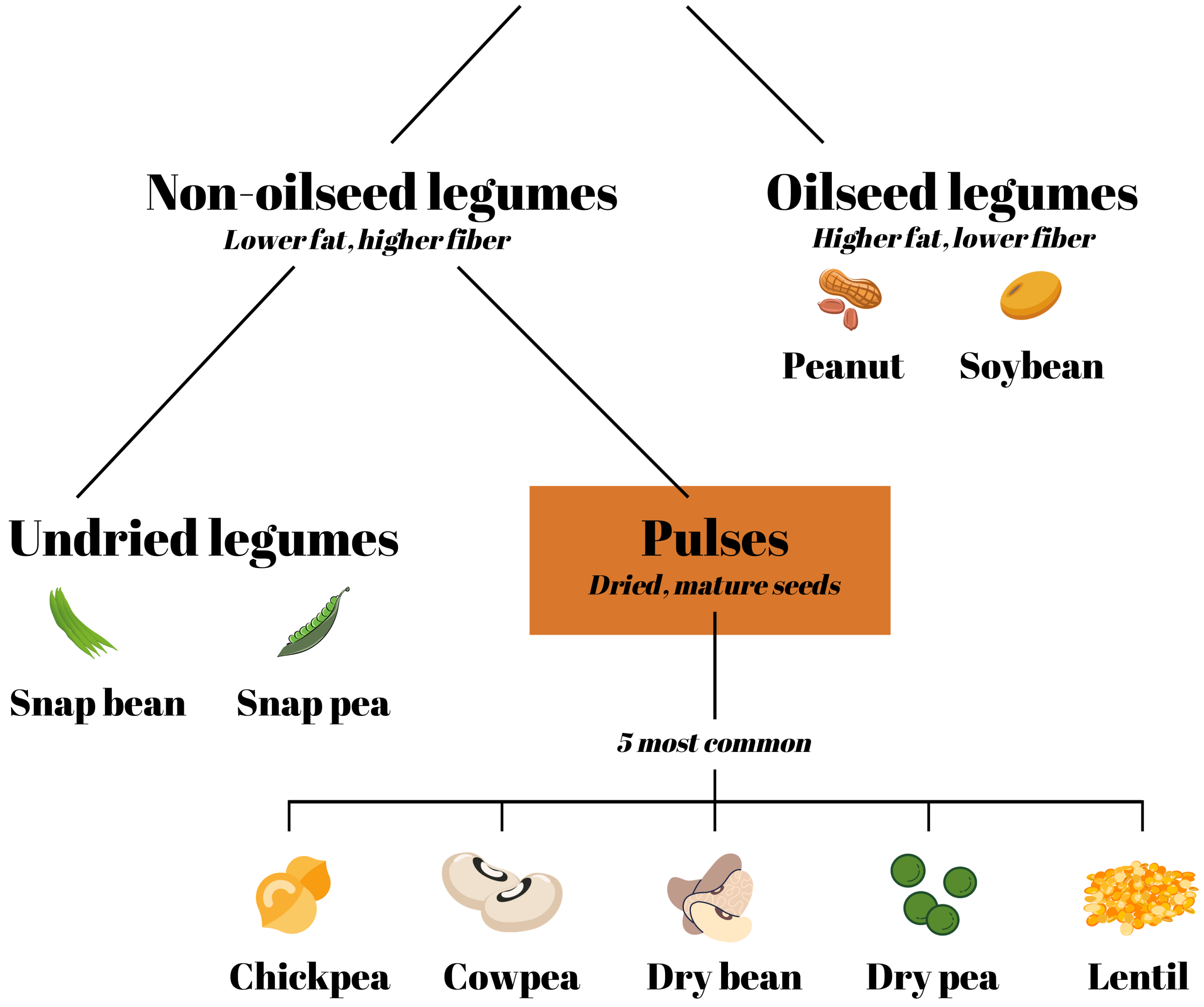

On a scale of 1 (low) to 5 (high), how would you rate your current knowledge (**after** taking the class) of the following?

	1 (low)	2	3	4	5 (high)
Knowledge of bean/pulse nutrition and health benefits	○	○	○	○	○
Knowledge of ways to use beans/pulses in various dishes	○	○	○	○	○

	1 (low)	2	3	4	5 (high)
Knowledge of how to prepare dry beans/pulses	○	○	○	○	○

As a result of the class, how likely are you to purchase and cook with **Colorado-grown** beans and other pulses?

- ○ Extremely likely
- ○ Somewhat likely
- ○ Neither likely nor unlikely
- ○ Somewhat unlikely
- ○ Extremely unlikely

As a result of the class, how likely are you to do the following?

	Extremely **likely**	Somewhat **likely**	Neither likely nor unlikely	Somewhat **unlikely**	Extremely **unlikely**
Eat more beans and other pulses	○	○	○	○	○
Use dry pulses instead of canned	○	○	○	○	○
Share something you learned today with someone else	○	○	○	○	○

What changes, if any, do you intend to make to how you cook dry beans and other pulses?

What information shared, if any, most motivated you to eat more pulses?

What new ways, if any, are you looking forward to including pulses in meals?

What did you find most interesting about the class?

Please share any feedback, suggestions, or comments about the class to help us improve.

Thank you for taking the time to fill out this post-survey!

To match your responses at the three time points, **please provide your email address**. Your responses will not be associated with your name or email - this is simply to help us match survey responses and know to whom we should send a thank you $10 Amazon gift card (available to the first 100 participants who complete all 3 surveys: the pre-survey, this post-survey, and the 1-month follow-up survey).

Powered by Qualtrics
