# Supplementary material for: A Translational Approach to Increase Pulse Intake and Promote Public Health through Developing an Extension Bean Toolkit
Source: Nutrients. 2023 Sep 24;15(19):4121. doi: 10.3390/nu15194121 (PMC10574132; doi:10.3390/nu15194121)
Supplement: Supplementary file 1 [file nutrients-15-04121-s001.zip › Supplementary Materials File S4. Extension validation class - 1-Month follow-up survey.pdf]

Default Question Block

Please take this follow-up survey approximately 1-month AFTER participating in the Colorado State University Extension pilot for the Beans: Good for You, Good for the Planet class. This online class is part of a PhD research project being conducted in the Horticulture and Landscape Architecture and Food Science and Human Nutrition Departments of Colorado State University to address topics a recent survey indicated are of interest, such as simple ways to regularly enjoy more beans, dry bean cooking tips, and the many health benefits of beans.

This survey should take about 5 minutes to complete. Your participation is voluntary, and you may skip any question you choose not to answer. You must be 18 or older to participate. Researchers will keep all information confidential. If you have questions, please contact PhD Candidate Chelsea Didinger, at Chelsea.Didinger@colostate.edu, or Dr. Marisa Bunning, Extension Specialist and Professor, at Marisa.Bunning@colostate.edu. If you have any questions about your rights as a volunteer in this research, contact the CSU IRB at: RICRO_IRB@mail.colostate.edu; 970-491-1553.

COLORADO STATE UNIVERSITY
EXTENSION

Your input is critical and appreciated, as this class and the associated surveys are important to the research project and will allow us to improve upon the class and continue to deliver it into the future. As a thank you for participating, the first 100 people who complete ALL 3 brief surveys (the pre-survey, the post-survey after the class, and this 1-month follow-up survey) will receive a $10 Amazon gift card.

Thank you for your valuable time and input – your contribution makes this research possible!

- ○ YES, I voluntarily agree to participate in this research.
- ○ NO, please exit me from this survey.

Block 1

Thank you for agreeing to participate in this survey. First, we would like to ask about your cooking and eating habits. Some of these questions may look familiar - that is part of the analysis.

Approximately how often do you eat beans or other pulses? Pulses include chickpeas, lentils, and dry peas like split peas but do NOT include soybeans, peanuts, snap peas, etc. See the graphic below for a more detailed definition of pulses.

- ○ Every day
- ○ 4-6 days per week
- ○ 1-3 days per week
- ○ 1-3 days per month
- ○ Several days per year, but less than 1 day per month
- ○ Never

Pulses are a type of legume that include dry beans like black beans, pinto beans, and kidney beans. Chickpeas, cowpeas (i.e. black-eyed peas), dry peas, and lentils are also pulses. Soybeans and fresh green vegetables such as snap beans and snap peas are NOT considered pulses.

9 Major Legumes

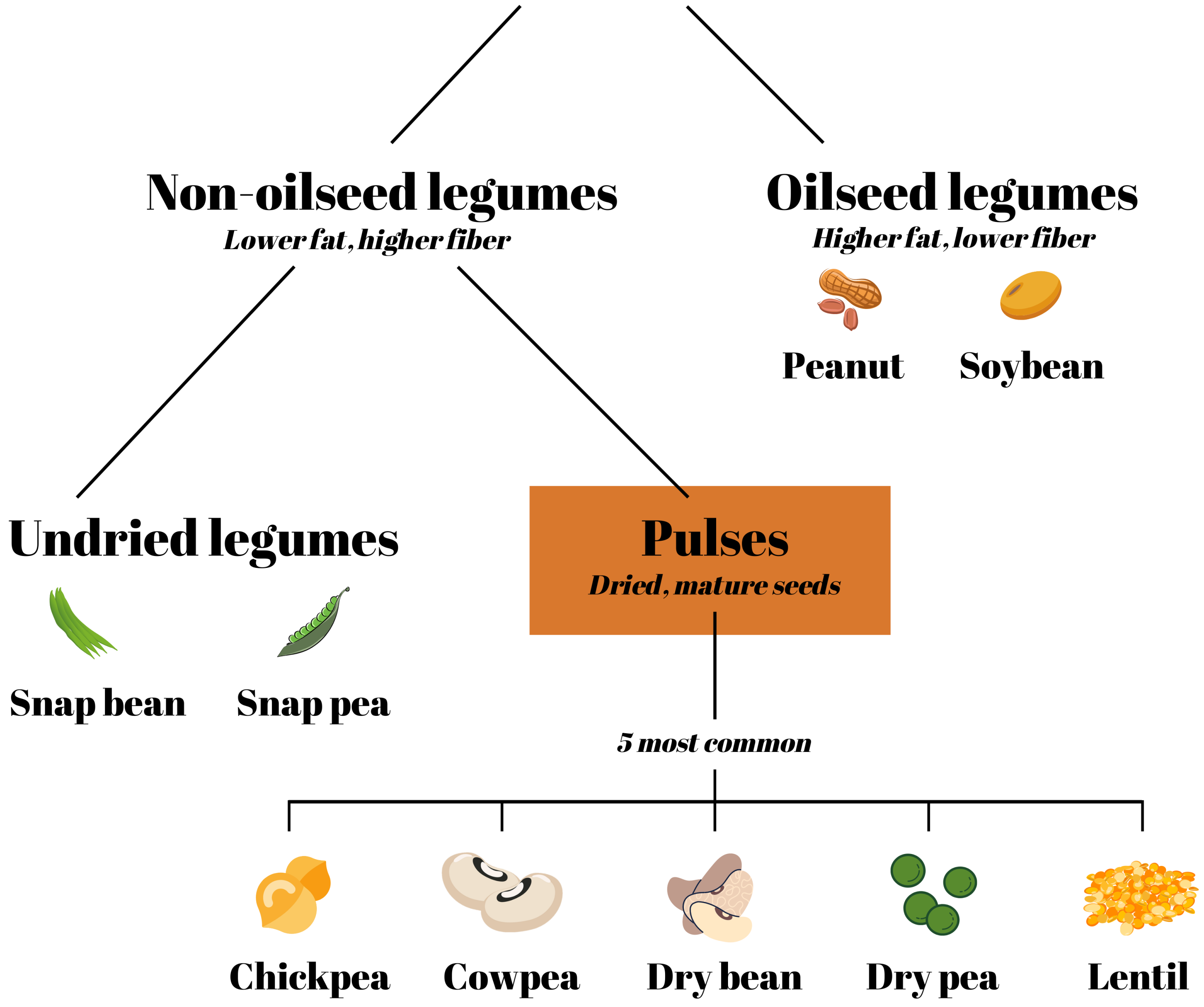

On a scale of 1 (low) to 5 (high), how would you rate your current knowledge of the following?

	1 (low)	2	3	4	5 (high)
Knowledge of bean/pulse nutrition and health benefits	○	○	○	○	○
Knowledge of ways to use beans/pulses in various dishes	○	○	○	○	○

	1 (low)	2	3	4	5 (high)
Knowledge of how to prepare dry beans/pulses	○	○	○	○	○

Which do you more frequently cook with, canned pulses or dry pulses you cook yourself?

- ○ Canned
- ○ Dry
- ○ Approximately 50-50

Approximately how often do you cook with **CANNED** beans or other pulses (including chickpeas, lentils, black-eyed peas, and split peas)?

- ○ Every day
- ○ 4-6 days per week
- ○ 1-3 days per week
- ○ 1-3 days per month
- ○ Several days per year, but less than 1 day per month
- ○ Never

Approximately how often do you cook using **DRY** (not canned) beans or other pulses (including chickpeas, lentils, and dry peas like split peas) cooked in the home?

- ○ Every day
- ○ 4-6 days per week
- ○ 1-3 days per week
- ○ 1-3 days per month
- ○ Several days per year, but less than 1 day per month
- ○ Never

As a result of the class, have you done any of the following? You can select multiple options.

- ☐ Cooked dry pulses for the first time
- ☐ Started cooking with dry pulses more often (as opposed to canned)

- [ ] Tried soaking dry pulses for the first time
- [ ] Added salt to the soaking water for the first time
- [ ] Tried cooking pulses in a different way (e.g., slow cooker, stovetop, electric pressure cooker) than you normally cooked them before the class
- [ ] Tried a new recipe or way to eat pulses (example: in a smoothie)
- [ ] Included more pulses in your eating pattern
- [ ] Shared information you learned with someone else

If you have tried a new way(s) to eat pulses since the class, please share what it was and what you thought.

As a result of the class, check the statement(s) with which you **agree**. You can select multiple options.

- [ ] I am more likely to try to source local, Colorado-grown beans
- [ ] I am more inclined to use dry beans instead of canned beans
- [ ] I am more likely to soak dry pulses before cooking
- [ ] I am more likely to add salt to the cooking water
- [ ] I am more likely to regularly eat beans and other pulses
- [ ] None of the above

Since the class, have you purchased Colorado-grown beans?

- ○ Yes
- ○ No
- ○ Unsure if the beans I purchased were from Colorado or not

What type(s) of Colorado-grown beans did you purchase? If possible, please share brand and type of bean.

Have you prepared the beans you purchased?

- ◯ Yes
- ◯ Not yet

What did you think about the Colorado-grown beans?

How likely are you to purchase local, Colorado-grown beans in the near future?

- ◯ Extremely likely
- ◯ Somewhat likely
- ◯ Neither likely nor unlikely
- ◯ Somewhat unlikely
- ◯ Extremely unlikely

Now, we would like to ask you one last time about factors that influence how often you eat beans and other pulses.

How important are the following nutritional aspects of pulses in **motivating** you to eat them?

	Very **important**	Somewhat **important**	Neither important nor unimportant (neutral)	Somewhat **unimportant**	Very **unimportant**
High fiber	◯	◯	◯	◯	◯
High protein	◯	◯	◯	◯	◯
Low fat	◯	◯	◯	◯	◯
Low calories	◯	◯	◯	◯	◯
Rich in some vitamins and minerals such as potassium and iron	◯	◯	◯	◯	◯

How important are the following reasons in **motivating** you to eat pulses?

	Very **important**	Somewhat **important**	Neither important nor unimportant (neutral)	Somewhat **unimportant**	Very **unimportant**
Human health benefits (may promote gut health and reduce risk for chronic diseases, etc.)	○	○	○	○	○
Environmental benefits/sustainability	○	○	○	○	○
Cost/affordability	○	○	○	○	○
Taste	○	○	○	○	○
Local	○	○	○	○	○

How important are the following in **discouraging** you from eating pulses? 'Important' reflects a factor that discourages you. 'Unimportant' represents a factor that does not discourage you.

	Very **important**	Somewhat **important**	Neither important nor unimportant (neutral)	Somewhat **unimportant**	Very **unimportant**
Gas/flatulence	○	○	○	○	○
Unsure how to prepare meals and/or snacks with pulses	○	○	○	○	○
Family and/or friends dislike eating beans and other pulses	○	○	○	○	○
Long cooking times	○	○	○	○	○
Dislike the taste	○	○	○	○	○

Is there anything else you would like to share?

If desired, please let us know of any particular topics you would like us to cover in future classes and/or materials.

Thank you for taking the time to fill out the final, 1-month follow-up survey!

To match your responses at the three time points, **please provide your email address**. Your responses will not be associated with your name or email - this is simply to help us match survey responses and know to whom we should send a thank you $10 Amazon gift card (available to the first 100 participants who complete all 3 surveys: the pre- and post-surveys and this 1-month follow-up survey). We will be in touch via email soon regarding your gift card if you are one of the recipients.

For gift card purposes, do you live in the United States?

- Yes
- No

Powered by Qualtrics
