# Supplementary material for: A Translational Approach to Increase Pulse Intake and Promote Public Health through Developing an Extension Bean Toolkit
Source: Nutrients. 2023 Sep 24;15(19):4121. doi: 10.3390/nu15194121 (PMC10574132; doi:10.3390/nu15194121)
Supplement: Supplementary file 1 [file nutrients-15-04121-s001.zip › Supplementary Materials File S5. Extension final class - Survey.pdf]

Default Question Block

Please take this survey after participating in the Colorado State University Extension class, Beans: Good for You, Good for the Planet. This online class is part of a PhD research project being conducted in the Horticulture and Landscape Architecture and Food Science and Human Nutrition Departments of Colorado State University to address topics a recent survey indicated are of interest, such as simple ways to regularly enjoy more beans, dry bean cooking tips, and the many health benefits of beans.

This survey should take about 5-10 minutes to complete. Your participation is voluntary, and you may skip any question you choose not to answer. You must be 18 or older to participate. Researchers will keep all information confidential. If you have questions, please contact PhD Candidate Chelsea Didinger, at Chelsea.Didinger@colostate.edu, or Dr. Marisa Bunning, Extension Specialist and Professor, at Marisa.Bunning@colostate.edu. If you have any questions about your rights as a volunteer in this research, contact the CSU IRB at: RICRO_IRB@mail.colostate.edu; 970-491-1553.

COLORADO STATE UNIVERSITY
EXTENSION

Thank you for your valuable time and input – your contribution makes this research possible!

- ○ YES, I voluntarily agree to participate in this research.
- ○ NO, please exit me from this survey.

Block 1

Thank you for agreeing to participate in this survey. First, we would like to ask about your cooking and eating habits. These questions relate to your current eating habits, not to any changes you intend to make after the class.

Approximately how often do you eat beans or other pulses? Pulses include chickpeas, lentils, and dry peas like split peas but do NOT include soybeans, peanuts, snap peas, etc. See the graphic below for a more detailed definition of pulses.

- Every day
- 4-6 days per week
- 1-3 days per week
- 1-3 days per month
- Several days per year, but less than 1 day per month
- Never

Pulses are a type of legume that include dry beans like black beans, pinto beans, and kidney beans. Chickpeas, cowpeas (i.e. black-eyed peas), dry peas, and lentils are also pulses. Soybeans and fresh green vegetables such as snap beans and snap peas are NOT considered pulses.

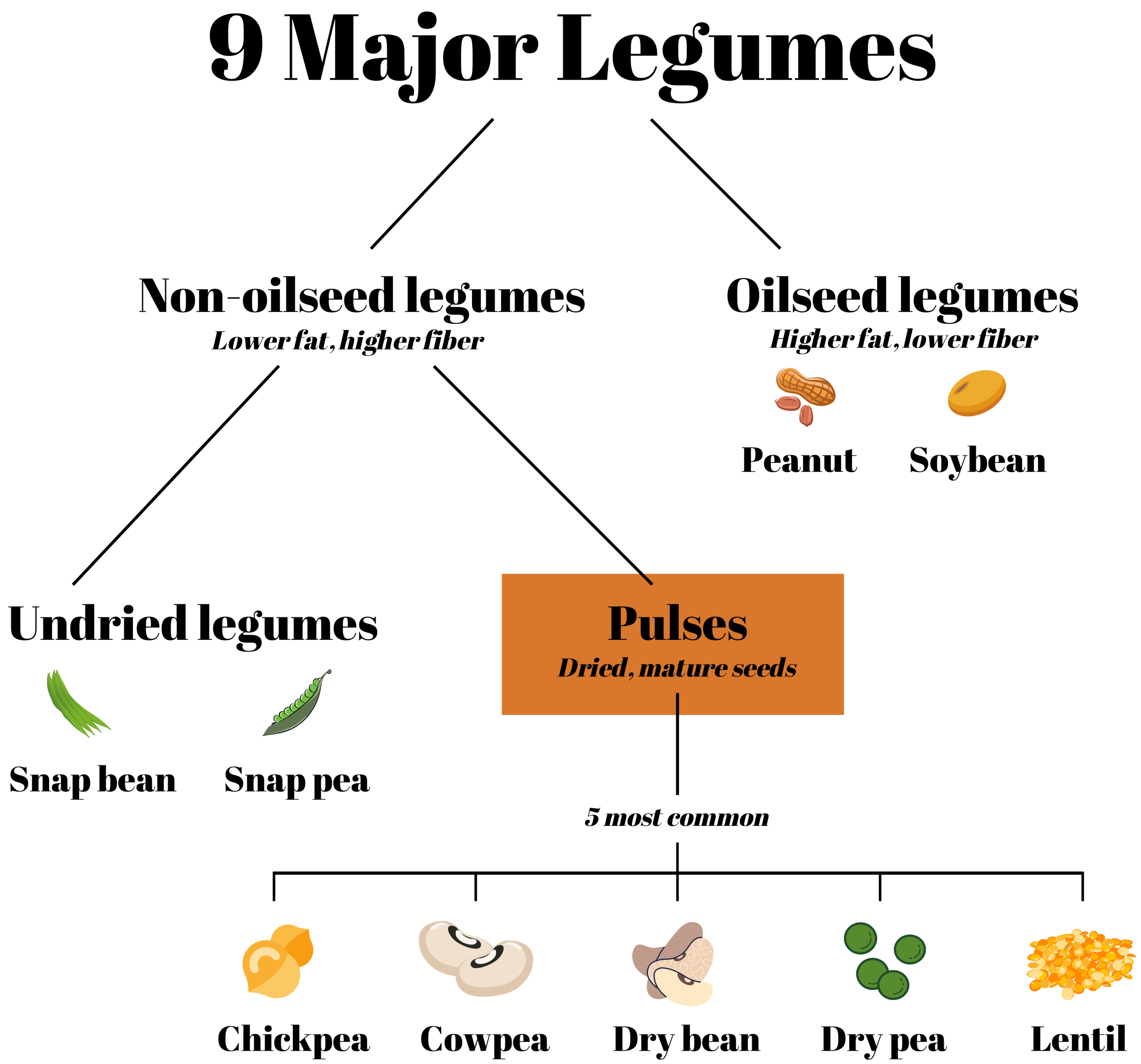

Which do you more frequently cook with, canned pulses or dry pulses you cook yourself?

- ○ Canned
- ○ Approximately 50-50
- ○ Dry

Approximately how often do you cook with **CANNED** beans or other pulses (including chickpeas, lentils, black-eyed peas, and split peas)?

- ○ Every day
- ○ 4-6 days per week
- ○ 1-3 days per week
- ○ 1-3 days per month
- ○ Several days per year, but less than 1 day per month
- ○ Never

Approximately how often do you cook using **DRY** (not canned) beans or other pulses (including chickpeas, lentils, and dry peas like split peas) cooked in the home?

- ○ Every day
- ○ 4-6 days per week
- ○ 1-3 days per week
- ○ 1-3 days per month
- ○ Several days per year, but less than 1 day per month
- ○ Never

BEFORE the class, approximately how often did you purchase and eat **Colorado-grown** beans and other pulses?

- ○ Never
- ○ Very rarely (1-20% of the time)
- ○ Somewhat rarely (21-40% of the time)
- ○ Sometimes (41-60% of the time)
- ○ Somewhat often (61-80% of the time)
- ○ Very often (81-100% of the time)
- ○ Unsure if the beans I purchase are from Colorado or not

Now, please share your feedback about the class.

Did you learn something new in the class?

- ○ Yes
- ○ No

On a scale of 1 (low) to 5 (high), how would you rate your knowledge of the following BEFORE the class?

	1 (low)	2	3	4	5 (high)
Knowledge of bean/pulse nutrition and health benefits	○	○	○	○	○
Knowledge of ways to use beans/pulses in various dishes	○	○	○	○	○
Knowledge of how to prepare dry beans/pulses	○	○	○	○	○

On a scale of 1 (low) to 5 (high), how would you rate your knowledge of the following AFTER the class?

	1 (low)	2	3	4	5 (high)
Knowledge of bean/pulse nutrition and health benefits	○	○	○	○	○
Knowledge of ways to use beans/pulses in various dishes	○	○	○	○	○
Knowledge of how to prepare dry beans/pulses	○	○	○	○	○

As a result of the class, how likely are you to purchase and cook with **Colorado-grown** beans and other pulses?

- ○ Extremely likely
- ○ Somewhat likely
- ○ Neither likely nor unlikely
- ○ Somewhat unlikely
- ○ Extremely unlikely

As a result of the class, check the statement(s) with which you **agree**. You can select multiple options.

- ☐ I am more likely to try to purchase local, Colorado-grown beans

- [ ] I now plan to try cooking dry beans for the first time (this only applies to people who have not cooked dry beans before)
- [ ] I am more inclined to use dry beans instead of canned beans
- [ ] I am more likely to soak dry pulses before cooking
- [ ] I am more likely to add salt to the cooking water
- [ ] I am more likely to regularly eat more beans and other pulses
- [ ] I am more likely to try a new recipe or way to eat pulses (example: in a smoothie)
- [ ] I am likely to share something I learned with someone else

What information shared, if any, most motivated you to eat more pulses?

What new ways, if any, are you looking forward to including pulses in meals?

What did you find most interesting about the class?

BEFORE the class, how important were the following nutritional aspects of pulses in **motivating** you to eat them?

	Very **important**	Somewhat **important**	Neither important nor unimportant (neutral)	Somewhat **unimportant**	Very **unimportant**
High fiber	○	○	○	○	○
High protein	○	○	○	○	○
Low fat	○	○	○	○	○
Low calories	○	○	○	○	○
Rich in some vitamins and minerals such as potassium and iron	○	○	○	○	○

AFTER the class, how important are the following nutritional aspects of pulses in **motivating** you to eat them?

	Very **important**	Somewhat **important**	Neither important nor unimportant (neutral)	Somewhat **unimportant**	Very **unimportant**
High fiber	○	○	○	○	○
High protein	○	○	○	○	○
Low fat	○	○	○	○	○
Low calories	○	○	○	○	○
Rich in some vitamins and minerals such as potassium and iron	○	○	○	○	○

BEFORE the class, how important were the following reasons in **motivating** you to eat pulses?

	Very **important**	Somewhat **important**	Neither important nor unimportant (neutral)	Somewhat **unimportant**	Very **unimportant**
Human health benefits (may promote gut health and reduce risk for chronic diseases, etc.)	○	○	○	○	○
Environmental benefits/sustainability	○	○	○	○	○
Cost/affordability	○	○	○	○	○
Taste	○	○	○	○	○
Local	○	○	○	○	○

AFTER the class, how important are the following reasons in **motivating** you to eat pulses?

	Very **important**	Somewhat **important**	Neither important nor unimportant (neutral)	Somewhat **unimportant**	Very **unimportant**
Human health benefits (may promote gut health and reduce risk for chronic diseases, etc.)	○	○	○	○	○
Environmental benefits/sustainability	○	○	○	○	○
Cost/affordability	○	○	○	○	○
Taste	○	○	○	○	○
Local	○	○	○	○	○

BEFORE the class, how important were the following in **discouraging** you from eating pulses?

	Highly discourages	Somewhat discourages	Minimally discourages	Very minimally discourages	Does not discourage
Gas/flatulence	○	○	○	○	○

	Highly discourages	Somewhat discourages	Minimally discourages	Very minimally discourages	Does not discourage
Unsure how to prepare meals and/or snacks with pulses	○	○	○	○	○
Family and/or friends dislike eating beans and other pulses	○	○	○	○	○
Long cooking times	○	○	○	○	○
Dislike the taste	○	○	○	○	○

AFTER the class, how important were the following in **discouraging** you from eating pulses?

	Highly discourages	Somewhat discourages	Minimally discourages	Very minimally discourages	Does not discourage
Gas/flatulence	○	○	○	○	○
Unsure how to prepare meals and/or snacks with pulses	○	○	○	○	○
Family and/or friends dislike eating beans and other pulses	○	○	○	○	○
Long cooking times	○	○	○	○	○
Dislike the taste	○	○	○	○	○

Please share any other feedback, suggestions, or comments about the class that you may have.

Last, please answer a few questions about yourself to help us better understand who we are serving with Colorado State University Extension programs.

In which Colorado county do you live? If you do not currently live in Colorado, please write 'N/A.'

Gender

- ◯ Female
- ◯ Male
- ◯ Other
- ◯ Prefer not to say

Do you identify as Hispanic?

- ◯ Hispanic
- ◯ Non-Hispanic
- ◯ Prefer not to answer

With which ethnicity do you most identify? You can choose multiple options.

- ☐ Asian
- ☐ Black
- ☐ Native American
- ☐ White/Caucasian
- ☐ Other
- ☐ Prefer not to answer

Age

- ◯ 18-20

- 21-29
- 30-39
- 40-49
- 50-59
- 60-69
- 70-79
- 80+
- Prefer not to say

Please provide your email address. This is just to ensure responses are not duplicated in the analysis, and your email will not be shared.

Powered by Qualtrics
