# Supplementary material for: A Translational Approach to Increase Pulse Intake and Promote Public Health through Developing an Extension Bean Toolkit
Source: Nutrients. 2023 Sep 24;15(19):4121. doi: 10.3390/nu15194121 (PMC10574132; doi:10.3390/nu15194121)
Supplement: Supplementary file 1 [file nutrients-15-04121-s001.zip › Supplementary Materials File S6. Food Habits Survey Demographics.pdf]

**Dietary Pattern**	***n* (out of 940)**	**Percent***
Omnivore	680	72.3
Pescatarian	106	11.3
Vegetarian	84	8.9
Vegan	69	7.3
Total responses	939	

**Gender**		
Female	751	79.9
Male	173	18.4
Other	3	0.3
Prefer not to say	10	1.1
Total responses	937	

**Age group**		
18-20	3	0.3
21-29	124	13.2
30-39	222	23.6
40-49	162	17.2
50-59	153	16.3
60-69	173	18.4
70-79	83	8.8
80+	6	0.6
Prefer not to say	13	1.4
Total responses	939	

**Hispanic**		
Hispanic	83	8.8
Non-Hispanic	814	86.6
Prefer not to answer	42	4.5
Total responses	939	

**Ethnicity**		
Asian	34	3.6
Black	26	2.8
Native American	36	3.8
White	778	82.8
Native American-White	6	0.6
Asian-White	9	1
Prefer not to answer	26	2.8
Black-Native American-White	3	0.3
Asian-Native American	3	0.3
Other	10	1.1
Total responses	931	

**Highest education level**	***n* (out of 940)**	**Percent***

Some high school, no diploma	7	0.7
High school graduate or GED	23	2.4
Some college credit, no degree	84	8.9
Trade/technical/vocational training	45	4.8
Associate degree	51	5.4
Bachelor’s degree, completed or currently enrolled	255	27.1
Master’s degree, completed or currently enrolled	309	32.9
Professional degree	62	6.6
Doctorate degree, completed or currently enrolled	85	9
Other	7	0.7
Prefer not to say	11	1.2
Total responses	939	
**Country**		
USA	839	89.3
UK	3	0.3
Australia	3	0.3
Canada	2	0.2
Other	6	0.6
Total responses	853	
**Region in the US**		
West Coast (CA, OR, WA)	211	22.4
Rocky Mountain (ID, MT, WY, CO, UT)	287	30.5
Southwest (NV, AZ, NM, TX)	45	4.8
Great Plains (ND, SD, NE, KS, OK)	17	1.8
Midwest (MN, WI, IA, MO, IL, IN, MI, OH)	67	7.1
South (AR, LA, MS, TN, KY, AL, GA, FL, SC, NC, VA, WV)	82	8.7
Mid-Atlantic (NY, PA, DE, MN, Washington DC, NJ, MD)	73	7.8
New England (VT, CT, RI, MA, NH, ME)	24	2.6
Non-contiguous states (AK, HI)	9	1
Reported multiple states	1	0.1
Total responses	816	
**Household Income Level**		
Less than $25,000	22	2.3
$25,000 - $49,999.99	97	10.3
$50,000 - $74,999.99	174	18.5
$75,000 - $99,999.99	134	14.3
$100,000 or greater	366	38.9
Prefer not to say	141	15
Total responses	934	

Percent out of n = 940 responses is shown, not valid percent
