# Supplementary material for: A Translational Approach to Increase Pulse Intake and Promote Public Health through Developing an Extension Bean Toolkit
Source: Nutrients. 2023 Sep 24;15(19):4121. doi: 10.3390/nu15194121 (PMC10574132; doi:10.3390/nu15194121)
Supplement: Supplementary file 1 [file nutrients-15-04121-s001.zip › Supplementary Materials File S7. Final Extension class participant demographics.pdf]

Gender	*n* (out of 86)	Percent*
Female	76	88.4
Male	10	11.6
Total responses	86	

Age group		
18-20	1	1.2
21-29	3	3.5
30-39	7	8.1
40-49	12	14.0
50-59	14	16.3
60-69	27	31.4
70-79	19	22.1
80+	2	2.3
Prefer not to say	1	1.2
Total responses	86	

Hispanic		
Hispanic	6	7.0
Non-Hispanic	79	91.9
Prefer not to answer	1	1.2
Total responses	86	

Ethnicity		
Asian	1	1.2
Native American	1	1.2
White	79	91.9
Other	1	1.2
Prefer not to answer	2	2.3
Native American-White and other ethnicity	1	1.2
Native American-Other	1	1.2
Total responses	86	

Live in Colorado?		
Yes	72	83.7
No	13	15.1
Total responses	85	

**Percent out of n = 86 responses is shown, not valid percent*
