# Supplementary material for: A Translational Approach to Increase Pulse Intake and Promote Public Health through Developing an Extension Bean Toolkit
Source: Nutrients. 2023 Sep 24;15(19):4121. doi: 10.3390/nu15194121 (PMC10574132; doi:10.3390/nu15194121)
Supplement: Supplementary file 1 [file nutrients-15-04121-s001.zip › Supplementary Materials File S8. Tables with Additional Participant Quotes.pdf]

Tables with Additional Class Participant Quotes

Below, find the tables that are displayed in the paper, but with additional participant quotes supporting the themes.

Table S1. * What information participants found most motivating and interesting about the Extension class

Theme	Motivator[1] – Example Quote	Interesting[2] – Example Quote
Health Benefits	• "Health benefits! I have high cholesterol and see that adding this fiber from pulses in as many ways as possible could be very beneficial for weight management and hopefully cholesterol reduction."<br>• "Fiber and protein content and gut health"<br>• "All the health benefits shared and the variety of recipes. I forget about beans sometimes but they are great!"<br>• "Their nutrition density is a big factor for eating more pulses as well as their versatility in cooking and over all health benefits including more satiety and weight loss :)"<br>• "The health benefits were more than I realized."	• "Significant benefits of consuming beans from the standpoint of both heart and gut health."<br>• "Understanding the health benefits and your gut will adjust to eating more beans."
Nutrition	• "The protein and fiber and potassium comparisons shared."<br>• "The comparison for protein and fiber content was really informative."<br>• "The stats on dietary fiber were quite eye-opening."<br>• "I didn't appreciate just how much more fiber they have than oats and other grains."<br>• "The nutritional piece. I knew they were good for me but not to that extent."<br>• "They are high in protein. I exercise frequently, and meeting my protein requirement is often a struggle."<br>• "I learned that pulses offer more nutrients than my usual breakfast of oat bran, so now it's 'beans for breakfast!' for me!"	• "The amount of fiber and protein in beans. I knew they were good for you, but I never really knew the exact reason why."<br>• "The entire class was interesting, but the fact about our dietary fiber gap really surprised me. Also that the pulses are so much higher in protein and fiber."<br>• "The most interesting fact that I learned in Chelsea's presentation is the high fiber and high protein content in pulses are essentially equal and they are considered a vegetable fiber source and a vegetable protein source, as well."
Environmental Benefits	• "Environmental value"<br>• "The tremendous health benefits for low cost, both monetary and environmental."<br>• "How good they are for the environment as compared to meat."	• "Interesting about drought tolerant which is more and more important."<br>• "Benefits to the soil and environment."
Affordable	• "Cost-very budget friendly."<br>• "Cheap, nutritious, easy to prepare different ways."<br>• "They are a cheaper source of protein."<br>• "I eat a lot of Fiesta, a frozen King Sooper/Kroger product. They have increased the price 69% since January! So I	• "That beans are a triple winner: highly nutritious, inexpensive, and easy on our environment."<br>• "The value proposition of beans...Much more versatile and nutritious than I realized."

	am starting to look for my preparation of chickpeas and other beans."	
Culinary Versatility	• "Thinking about how to add beans in food not otherwise typical."<br>• "I found the health benefits interesting as well as the several ideas for ways to eat more pulses."<br>• "Recipe suggestions. I get in a routine and don't try new recipe ideas, so this will be fun."<br>• "I appreciated the information and ideas of adding beans to a smoothie or making a bean dip. So many fun new ways to incorporate getting them into my diet."<br>• "The varieties of beans available."<br>• "The creative ways to add pulses to dishes and the beautiful and very appetizing pictures of pulses in dishes."<br>• "Use of pulses for every meal. Going to personally challenge myself to incorporate beans into breakfast and smoothie options."<br>• "I like the idea of mixing beans and meat to in-crease nutritional value. For example turkey burger mixed with bean"	• "I had never heard of aquafaba so will be fun to learn how to use this liquid and see if it whips up."<br>• "Aquafaba!!! I had no idea this was a thing or that it can be whipped like a meringue."<br>• "Suggestions on how to incorporate beans in more foods."<br>• "So many different uses for beans and a reminder to use them in salads!"<br>• "The many different ways they can be used, not just baked beans!"<br>• "Using bean flour."<br>• "I learned about the Mayocoba beans. I had never heard of them before and saw that they were at Walmart so will see if I can find them."<br>• "The yellow beans from Colorado. Have never seen them or tried them"<br>• "All the different textures/taste of beans"<br>• "Versatility is endless with beans."
Local	• "I had no idea that Colorado grew and produced so many beans!"<br>• "That there are a wide range of pulses grown locally."	• "The variety and amount of beans grown locally in Colorado. I'll keep an eye out for Colorado pulses."<br>• "That Colorado is the 6th highest bean-producing state."<br>• "I didn't realize Colorado grew so many beans and they can be purchased locally. I'll look into those. I also want to try the Mayocoba beans, these are new to me."
Presenter Enthusiasm	• "Honestly Chelsea's enthusiasm and passion alone was totally motivating to eat more beans!"<br>• "Enthusiasm of presenter and others in the class."<br>• "Chelsea's enthusiasm, makes me want to try more beans."	• "The speaker was very knowledgeable and personable. She is clearly passionate about the subject, and she was able to convey that enthusiasm to the audience."<br>• "The presenter was marvelous! She was very upbeat and engaging...and knowledgeable. I bet she could get anybody to eat beans!"<br>• "The presenter's interest and excitement about beans… who knew beans could be so interesting?"<br>• "Chelsea's enthusiasm for beans is contagious and it got me really excited."<br>• "The enthusiasm of the speaker - seriously, makes me want to buy bean and get cooking!"

[1]Responses to the question, "What information shared, if any, most motivated you to eat more pulses?"

[2]Responses to the question, "What did you find most interesting about the class?"

*Corresponds to Table 10 in the manuscript

Table S2. * New ways in which class participants looked forward to trying and/or did try pulses

Type of Dish	Looking Forward To[1] – Example Quote	New Dish They Tried[2] – Example Quote
Smoothies	• "Adding to smoothies! I love smoothies, but no matter how healthy I try to make them, it always feels like a smoothie will spike my blood sugar level. I think this will be an excellent way to mediate that."<br>• "My kids love smoothies so we'll add to smoothies."<br>• "Definitely interested in trying in smoothies, using as a main protein."<br>• "Me and those in my household very open to trying pulses in smoothies!"	• "I added beans to a smoothie. It was great! I shared that with others who said they would be interested in trying that as well."<br>• "Smoothies, I would have never thought to use them this way."
Pulse Products	• "Bean based pasta"<br>• "I like the idea of the chickpea flour for baking, too."<br>• "Pasta. Whole wheat pasta is not very good. Just discovered chickpea pasta and will try it.	• "Started buying chickpea chips instead of corn chips."<br>• "My husband and I tried chickpea pasta and a protein+ pasta (containing lentil, pea, chickpea, wheat and barley flours, instead of the traditional white pasta we get. It was really good! Much better than the whole wheat pasta."
Baking & Desserts	• "I like the idea of the chickpea flour for baking, too."<br>• "I love to bake, I would like to try baking recipes."<br>• "Black bean brownies sounded very interesting."<br>• "The idea of making desserts more nutritious has got me inspired."<br>• "I will try to include beans in my diet more and in baking! I made black bean brownies for the first time right after the talk."	• "Black bean brownies. Loved them!"<br>• "Black bean chocolate mousse. Delicious, dense and filling."
Combining with Meat	• "Half and half when we would typically use all beef."<br>• "Adding them more to meat dishes."	• No quote received
Breakfast	• "Breakfast! Never considered that"<br>• "I am really going to try and eat a lot more beans, and incorporate them into more breakfasts and lunches since I just have had them mainly for dinners and in soups."<br>• "I have never thought about having beans for breakfast so I will give that a try soon"	• "Pulses in breakfast foods was new to me. I'm still getting used to the texture. It's a bit heavy."
Adding to Favorite Dishes	• "I want to try some of the recipes that were shown and try adding pulses to meals we already eat."<br>• "Adding beans to meals we already make."<br>• "I plan to toss them into whatever I make."	• "Before the class I only ate them as a side dish or in chili or bean soup. I tried making a bean dip and it worked but I'm too lazy for that. Now I just try add a bit to every lunch and dinner. For instance I add them to my salads whether that be a meal salad or a side salad. Or when I

		have steak, I make the steak a smaller portion and replace that portion with beans sprinkled with steak seasoning."<br>• "Added them to a meal that didn't include them originally."
Salads	• "More use in salads!"<br>• "Adding beans to salads. I love one meal salads!"	• "Added canned chickpeas to a salad-- very good."
Dips	• "I really like hummus and think that it will be easier to incorporate it into more meals as a start."<br>• "Easy - first thing I'm going to try: Olive-y Bean Dip!!! YUM!"	• "I tried the spread with the olives, and it was delicious!"<br>• "Made hummus with black beans."
Aquafaba	• "Use bean liquid (forgot about the term) for cooking rice and soups."<br>• "Using cooking water for egg whites,"<br>• "I want to try whipping bean broth. Will use bean broth in making soup."	• "Used the bean cooking water in a smoothie."
With Other Grains & Carbohydrates	• "The pulses mashed with avocados on toast sounded really good."<br>• "Adding them to pasta"<br>• "Combing them with mashed potatoes"	• "I have put in with my oatmeal!"
New Pulse Varieties	• "Try the Mayocoba beans. Never heard of them before."<br>• "Honestly I bought a bunch of beans and am looking forward to trying them all in different ways!"<br>• "More creative thinking to include more beans. Experimentation with additional varieties."	• "I tried northern beans for the first time! Loved them."

[1]Responses to the question, "What new ways, if any, are you looking forward to including pulses in meals?"
[2]For those who participated in the 1-month follow-up survey for the validation class, responses to the question, "If you have tried a new way(s) to eat pulses since the class, please share what it was and what you thought." The responses do not line up, i.e., responses of what participants actually tried have not been matched to what they indicated they are interested in trying, as these responses came from both the validation and final class and multiple surveys.
*Corresponds to Table 11 in the manuscript

Table S3. * Intended changes to pulse intake and preparation habits

Theme	Example Quote
Intake	• "Try to incorporate pulses into daily life!"<br>• "I obviously need to eat way more and make them a regular part of my diet."<br>• "Your presentation was a reminder to use pulses more in my cooking. I've eaten beans, in some form, every day since your talk. Can't promise that trend will continue, but I hope that eating them more often becomes a permanent habit."<br>• "I would like to start the journey towards 2 c. of pulses each day."<br>• "Found the presentation very informative and am definitely incorporating more pulses into my diet."<br>• "I intend to cook and eat beans more regularly. At least twice a month vs. a few times a year."<br>• "Now that this class encouraged and reminded me about slow cooker and pressure cookers, that I already have, plus that I can freeze them I'll be eating beans with just about every meal."
Use Variety	• "Look into other options when needing something that cooks more quickly (lentils instead of black beans)."<br>• "I enjoyed the presentation and am encouraged to try many new ways. I had never thought of adding white beans to potatoes, macaroni and kidney beans, bean toast, black beans to a chocolate smoothie, or to egg scrambles or breakfast burrito. My mindset before had been just have soups with beans, add beans to taco salads or as a side dish. So many more choices now so thank you!"
Cooking Dry Pulses	• "I've never cooked dry beans so I'm very excited to try."<br>• "I will try to use more dry beans instead of can."<br>• "Using more of dry pulses"
Soaking	• "Adding salt to soaking!"<br>• "Will soak beans to reduce flatulence."<br>• "I have never tried quick soak method shared in the class today so will give it a try also."
Cooking Method	• "The slow cooker seems like a good option to try."<br>• "I will try other cooking methods."
Freezing	• "Can cook enough to freeze and have on hand quickly."<br>• "I also like the idea of saving time by cooking lots and freezing it."

* Corresponds to Table 12 in the manuscript
